# High levels of flame retardants in vehicle dust indicate ongoing use of brominated and organophosphate flame retardants in vehicle interiors

**DOI:** 10.1007/s10661-025-13822-z

**Published:** 2025-03-15

**Authors:** Petra Svobodová, Simona Rozárka Jílková, Jiří Kohoutek, Ondřej Audy, Petr Šenk, Lisa Melymuk

**Affiliations:** RECETOX, Faculty of Science, Masaryk University, Kotlářská 2, 611 37 Brno, Czechia

**Keywords:** Dust exposure, Flame retardants, PFAS, Time trends, Cars

## Abstract

Vehicles are unique indoor environments, with interiors dominated by plastic/synthetic materials and exposure to extremes of temperature and radiation, leading to substantial potential for emissions of plastic additives from vehicle materials and subsequent exposure to drivers and passengers. Flame retardants (FRs) and per- and polyfluoroalkyl substances (PFAS) were measured in 30 dust samples collected from dashboards, seats, and trunks of cars of the same make and model (year of manufacture 1996–2021) to evaluate levels in dust and time patterns in additive use across cars of different ages. PFAS were detected in all dust samples at low levels, while FRs were detected in all samples, with some compounds consistently exceeding µg/g levels, especially tris(1,3-dichloro-2-propyl) phosphate (TDCIPP) and decabromodiphenyl ether (BDE-209), substantially higher than in other indoor environments. Although cars were of the same model, large variations were observed in FR concentrations in dust between cars, emphasizing the challenge in generalizing FR exposures from vehicle dust. Concentrations of BDE-209 in vehicle dust did not decrease over the 1996–2021 period, suggesting that restrictions on DecaBDE have had limited impact, likely due to exemptions in regulations for the automotive industry. The high FR levels indicate ongoing use of both organophosphate and brominated FRs in vehicles on the European market, although flammability standards for interior car materials are not mandated by European regulations, and the continued presence of long-restricted FRs suggests the presence of recycled plastics in vehicles; this potential exposure source may be increasing as vehicle producers aim to improve material circularity.

**Supplementary Information:**

The online version contains supplementary material available at 10.1007/s10661-025-13822-z.

## Introduction

Vehicles are a unique indoor environment because their interiors are almost entirely composed of plastic/synthetic polymer-based materials, which plays a significant role in the introduction of compounds of concern, including flame retardants (FRs), perfluorinated and polyfluorinated substances (PFAS), and plasticizers, into the vehicle environment. Moreover, vehicles are exposed to more extremes of temperature and radiation than other indoor environments, leading to potential increases in emissions from materials in vehicles and greater potential for degradation of polymer components (Hoehn et al., 2024; Reddam & Volz, 2021).

PFAS are used as surfactants, stain and water repellents, and surface protectors (Glüge et al., 2020), and are associated with cleaning products, including car care products (Glüge et al., 2020), and stain-resistant upholstery, including textiles like car seats and floor covers (Bečanová et al., 2016; Dewapriya et al., 2023; Tolaymat et al., 2023), and have been linked with adverse immune outcomes, cancer and neurodevelopment (Sunderland et al., 2019). FRs are widely used in car electronics (Sugeng et al., 2018), textiles, and seats (Shindo et al., 2024; Wu et al., 2019) to meet flammability standards. From the 1970s to the early 2000s, polybrominated diphenyl ethers (PBDEs) were the dominant FRs used (Alaee et al., 2003), but due to concerns about negative environmental and health effects, these compounds were phased out/banned over two decades, culminating in a ban by the Stockholm Convention in 2019, with exceptions remaining for the aerospace and vehicles industries until 2036 (Secretariat of the Stockholm Convention, 2024a, b). PBDE exposures have been associated with negative neurodevelopment, behavioral, and reproductive effects (Herbstman et al., 2010; Lyche et al., 2015). However, alternative FRs, novel halogenated flame retardants (NHFRs) and organophosphate esters (OPEs), are not without concerns; Dechlorane Plus was added to the Stockholm Convention in 2023, and some OPEs have endocrine-disrupting potential, specifically anti-androgenicity, and have been associated with allergic disorders and cancer (Bajard et al., 2021; Blum et al., 2019; Van Der Veen & De Boer, 2012; Wei et al., 2015).

Road vehicle standards differ between passenger vehicles, buses, and transport vehicles; for example, in the EU, flammability is regulated for long-distance buses through Regulation (EU) 2019/2144 (European Commission, 2019), but there are no binding regulations for passenger cars that address the flammability of interior materials. Globally, vehicle standards are governed by the World Forum for Harmonization of Vehicle Regulations of the Inland Transport Committee of the United Nations Economic Commission for Europe (UNECE), which has 58 parties, extending well beyond Europe, and the regulations of this body are generally recognized by non-signatories, except for the USA and Canada (UNECE, 2024a, b). UNECE has regulations covering flammability in vehicles, e.g., UNECE R118 on flammability of materials in buses, UNECE R34 on fire risks of fuel systems, and UNECE R100 on electric vehicles (UNECE, 2016, 2024c). However, while fuel systems and battery components in passenger cars are covered, global flammability standards for most interior materials are not mandated. In contrast, the USA has Federal Motor Vehicle Safety Standards (FMVSS), of which FMVSS 302 (Office of the Federal Register, 2023a) has requirements for the burn resistance of interior materials in motor vehicles, and FMVSS 305 (Office of the Federal Register, 2023b) covers electric vehicles. Canada has a system of analogous rules harmonized with US FMVSS (Legislative Services Branch, 2020). In China, the flammability standards for automotive interior materials are primarily governed by Guobiao 8410–2006 (MPR China Certification GmbH, 2024), and also largely align with US FMVSS 302. Many major vehicle producers align with American standards through their own internal standards and testing guidelines (e.g., Volvo VCS 5031.19, GM/Opel GMW 3232) even for vehicles produced and marketed outside of North America and China, which is expected to lead to the inclusion of chemical FRs in multiple vehicle interior parts.

Numerous studies have identified higher levels of plastic additives in vehicle dust than in other indoor microenvironments, and exposure to vehicle air (particularly with high volatile organic compound content) has been associated with negative health impacts (Liang et al., 2019). Studies have reported higher levels of flame retardants in cars than in homes (Abdallah & Covaci, 2014), e.g., concentrations of BDE-209 in car dust up to 35,500 ng/g and OPEs up to 109,000 ng/g (Ali et al., 2016). Longer commutes have been associated with higher exposure to OPEs (Reddam et al., 2020). Recent work has identified unique bioactivity in vehicle dust, particularly high anti-androgenicity in car dust that was not observed in any other indoor microenvironment (Pinto-Vidal et al., 2024).

Despite evidence of high exposure to synthetic compounds in vehicles, systematic evaluation is lacking. Studies often evaluate different components of vehicle dust (e.g., seat dust vs. dashboard dust) (Ali et al., 2011), and cover vehicles of different ages and models (Abdallah & Covaci, 2014; Gevao et al., 2016; Harrad et al., 2016a). This has reduced our ability to identify clear patterns, time trends or dominant compounds in vehicle dust. In this study, we select a subset of vehicles of the same brand and models and quantify > 50 flame retardants and PFAS (Table S1) in dust from different areas of the vehicles to evaluate spatial variations in additives in dust within vehicles and time patterns in additive use across similar cars of different ages. We support this specific analysis by evaluating the data in the context of the existing literature on FRs in vehicle dust to identify if such patterns can be more broadly observed.

## Material and methods

### Sampling

Previous studies have hypothesized that some of the large variability in concentrations of plastic additives in vehicle air and dust is due to the collection of samples from different makes and models (Lagalante et al., 2009). To eliminate this variability, to support the identification of time trends, we selected vehicles from two European market models of a single manufacturer (Škoda Octavia and Škoda Fabia), both of which have their final assembly within Europe, and a large part of the suppliers from the Czech Republic and Germany (Schäfer, 2021; Škoda Auto, 2021). We sampled five Škoda Octavia and five Škoda Fabia in May 2021 in Brno, Czechia. The cars ranged in age of manufacturing from 1996 to 2021 (Table S2).

In each car, three separate samples of settled dust were collected from (1) dashboard, (2) seats, and (3) trunk using a Hoover Telios 2300W vacuum cleaner with dust sampling head and a holder for a quartz fiber filter (QFF) on which the dust was captured (Fig. S1). A pre-weighed QFF was used for dust collection (QMA, 101.6 mm, 1851–101 Whatman). For collection on seats and in the trunk, a stainless-steel sampling head was used (Fig. S2). Unfortunately, this sampling head was too large to access dashboard spaces, therefore a separate plastic dashboard cleaning vacuum attachment was used (Fig. S3). After collection, filters were wrapped in aluminum foil in a sealable airtight bag and stored at − 18 °C until processing. At the time of sampling, a short questionnaire was administered to car owners to gather information about car use and cleaning habits (Table S3). Data were anonymized.

### Extraction and clean-up

Extraction and clean-up procedures are given in detail in the SI (Text S1). Before extraction, the filters containing the dust samples were ground using a ball mill and kept at − 4 °C between processing steps.

A 20% aliquot by mass was taken for the extraction and analysis of OPEs and PFAS. The remaining 80% of the aliquot was used for the extraction and analysis of PBDEs and NHFRs. The 20% aliquot was spiked with surrogate standards (Table S4) and extracted using methanol via ultrasonic extraction. The extract was concentrated under nitrogen and stored at 4 °C until analysis. The 80% aliquot was spiked with surrogate standards (Table S4) and extracted using 1:1 hexane–acetone via ultrasonic extraction. The extract was concentrated under nitrogen, then divided by weight into 30% and 70% fractions, and further concentrated. The 30% fraction, used for analysis of NHFRs, was purified with an activated silica gel column and eluted with hexane, followed by a rinse with dichloromethane. The 70% fraction, used for analysis of PBDEs, was purified on sulfuric acid-modified silica gel and activated silica gel, eluted with hexane:dichloromethane. Nonane was added to both fractions as a final solvent, and extracts were concentrated under nitrogen flow to a final volume of 50 µl. Internal standards (Table S4) were added, and samples were stored at 4 °C until analysis.

The dust extracts were analyzed for 10 PBDEs, 16 PFAS, 20 NHFRs, and 13 OPEs (Table S1) using previously validated methods. The instrumental methods are given in detail in the SI (Text S2).

### QA/QC

To track the accuracy of the analytical methods, three replicates of NIST Standard Reference Material® (SRM®) 2585 were processed with the same methods as the samples. Measured concentrations were compared with certified values NIST SRM® 2585 (NIST, 2005) or other studies that reported concentrations (Fan et al., 2014; Reiner et al., 2015). Most compounds showed good agreement with the certified values (Figs. S4–S6). Only two OPEs, triphenyl phosphate (TPhP) and tributyl phosphate (TBP), had somewhat lower values in our data than the certified/published values (Fig. S4) but we included them in further analysis with the caveat of possible underestimation of dust concentrations.

For decabromodiphenyl ethane (DBDPE) in a subset of samples, because of issues with sensitivity and high concentrations in the dust, the internal standard could not be used for quantification. In these cases, we used both a 4-point external calibration and retention factor methods to estimate concentrations, and took the average of both methods as an indicative DBDPE concentration. These values therefore have higher uncertainty than the DBDPE values obtained by internal standard. The values for which this external calibration method was used are indicated in Table S10.

Because vehicles were sampled in the parking area of the analytical laboratories, no travel blanks were collected. Three filter blanks and three solvent blanks were processed with the samples. Filter blanks were collected by inserting and then removing a clean filter into the sample collection head. Method detection limits (MDL) were determined based on filter blank masses and were calculated as the average of the blanks + 3*standard deviation of the field blanks. If all three blanks were below instrument detection levels, the instrument detection limit was used as the MDL. For statistical analyses, 0.5*MDL was substituted for samples that were < MDL. Samples > MDL were corrected for blank contamination by subtracting the average of the filter blanks. Blank masses are given in the SI (Text S3, Table S5).

Statistical analyses were performed using non-parametric tests (e.g., Mann–Whitney *U* tests), with a significance level set at *p* < 0.05.

## Results

### Detection and concentrations in vehicle dust

**Fig. 1:**
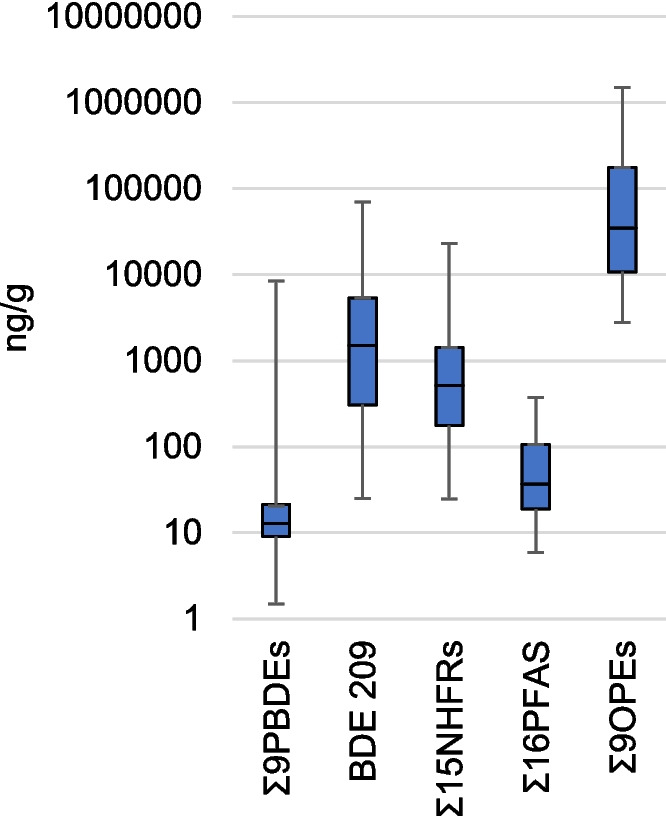
Ranges of compounds detected in the 30 car dust samples. The box shows the median and 25th/75th percentiles while the whiskers indicate the max–min values

Five PFAS were detected in > 90% of the car dust samples: perfluorooctanoic acid (PFOA), perfluorononanoic acid (PFNA), perfluorodecanoic acid (PFDA), perfluorododecanoic acid (PFDoDA), and perfluorotetradecanoic acid (PFTeDA). PFAS were at relatively low concentrations in the dust, typically in the ng/g range. PFOA was detected at the highest concentrations, with a median of 9.08 ng/g, followed by perfluorooctanesulfonic acid (PFOS) with a median of 5.28 ng/g, and PFDA with a median of 3.70 ng/g (Fig. 1, Tables S6–S7).

**Fig. 2:**
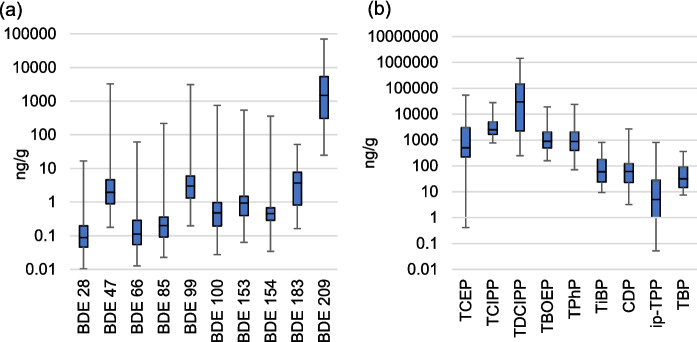
Ranges of **a** PBDE and **b** OPE concentrations in car dust. The box shows the median and 25th/75th percentiles while the whiskers indicate the max–min values. Compound abbreviations can be found in Table S1

PBDEs were consistently detected in all dust, with large differences in concentrations between BDE-209 and the other lower brominated PBDE congeners (Fig. 1, Tables S8–S9). BDE-209 was the third highest concentration chemical detected in car dust (after tris(2-chloroisopropyl)phosphate—TCIPP and tris(1,3-dichloro-2-propyl) phosphate—TDCIPP), with a median concentration of 1500 ng/g, and a maximum of 70,300 ng/g in dust from a 2009 car, a value exceeded only by TDCIPP. The presence of BDE-183, BDE-99, and BDE-47 was also noted, with median concentrations of 3.70 ng/g, 3.04 ng/g, and 1.97 ng/g, and maxima of 51.6 ng/g, 3120 ng/g, and 3290 ng/g (Fig. 2a), respectively, in dust from the 1996 car.

The highest levels of NHFRs were found for bis(2-ethylhexyl) tetrabromophthalate (BEH-TEBP), with a median of 175 ng/g, and a maximum of 14,100 ng/g in 2002 car dust. High levels were also noted for DBDPE, with a median of 60.8 ng/g, and 2-ethylhexyl 2,3,4,5-tetrabromobenzoate (EH-TBB) 8.50 ng/g. DDC-CO, α + β + δ + γ−1,2-Dibromo-4-(1,2-dibromoethyl)cyclohexane (ΣDBE-DBCH), 1,2-bis(2,4,6-tribromophenoxy)ethane (BTBPE) and pentabromotoluene (PBT) medians also exceeded the ng/g level. Other NHFRs were detected with medians of 0.07–0.67 ng/g (Tables S10–S11).

TCIPP, TDCIPP, tris(2-butoxyethyl) phosphate (TBOEP), triisobutyl phosphate (TiBP), TPhP, and TBP were detected in 100% of samples. OPE concentrations generally exceeded those of other compounds (Fig. 1). The highest concentrations were found for TDCIPP, with a median of 29,800 ng/g and a maximum of 1,430,000 ng/g, followed by TCIPP with a median of 2,500 ng/g, TBOEP with 900 ng/g, TPhP with 887 ng/g, and tris(2-chloroethyl) phosphate (TCEP) with 503 ng/g. Other OPEs were generally below 100 ng/g (Fig. 2b, Tables S12–S13).

**Fig. 3:**
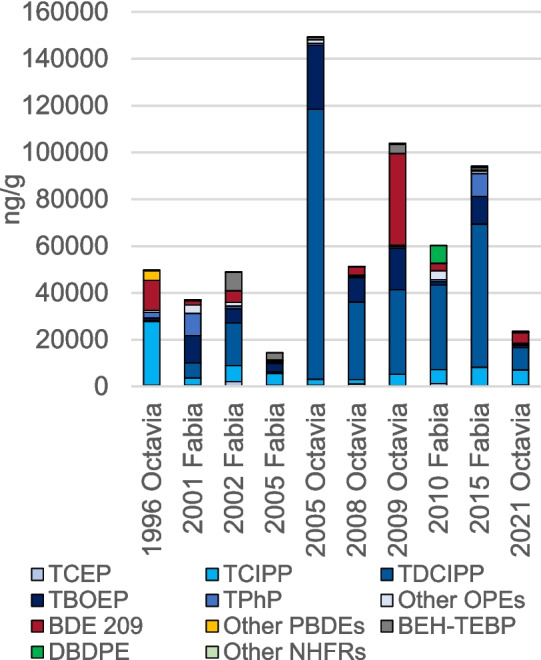
Composition of FRs in average of dashboard and seat dust from individual vehicles

Most compounds spanned large ranges across vehicles, typically 3–4 orders of magnitude (Fig. 1), despite all vehicles being of only two models from the same vehicle producer and all sold on the Czech market. This demonstrates the challenge in trying to generalize levels and composition of FRs or other synthetic chemicals in vehicle dust and understand patterns in their use. Most vehicle dust had a high burden of FRs; the sum of the 30 FRs included in our study ranged tenfold, from 14,000 to 140,000 ng/g, and compositions were similarly variable, even in vehicles of the same type produced at similar times (Fig. 3). While some variability can be attributed to differences in vehicle use, such large variations suggest differences in the additive content of the vehicle parts.

### Variation in dust content within a vehicle

#### PFAS

The highest concentrations of PFAS were consistently detected in dust from the dashboard area. All PFAS except for PFOS and perfluorohexanesulfonic acid (PFHxS) had significantly higher concentrations in dashboard dust compared to other car parts (Mann–Whitney U test, *p* < 0.05, Table S14). For example, PFOA had a median concentration of 30.3 ng/g on the dashboard, while the seats and trunk had 4.07 ng/g and 6.95 ng/g, respectively; PFDA and PFDoDA had median concentrations of 14.4 ng/g and 13.5 ng/g on the dashboard, while concentrations in the seats and trunk were 1–3 ng/g (Table S14). For PFOS, the values did not differ between individual parts. The concentrations on the seats and the trunk did not differ significantly for any PFAS.

These spatial differences suggest the dashboard to be the main source of PFAS in the dust, and by extension, the area likely associated with most PFAS use. We hypothesized that the contribution may be from the use of PFAS in dashboard protection sprays and residues from manufacturing, as PFAS compounds can be used as release agents in plastic moulding, although there is also a large set of other possible PFAS uses (Glüge et al., 2020). The generally low PFAS levels in the vehicles could be a consequence of car owner habits: according to a questionnaire of car owners, only two had used dashboard cleaning sprays, but neither of these cars had elevated PFAS levels in dashboard dust, suggesting the sprays did not contain PFAS on our target list. The PFAS content of many products used in car interiors is not disclosed, thus car owners are unlikely to know whether a protecting spray used on car interiors includes PFAS compounds. We also hypothesized that the presence of child car seats could lead to higher levels of PFAS in dust, but this effect was not seen.

We note that while PFAS levels were low, new thresholds for PFAS residues in products are also low, e.g., the proposed limit of 25 ppb for individual PFAS in the REACH PFAS restriction proposal (ECHA, 2023), and the presence of median PFOA concentrations in dashboard dust at levels above this threshold suggests possible PFOA use in vehicles at levels that could be non-compliant in future.

#### PBDEs

Concentrations of BDE-47, BDE-99, BDE-100, and BDE-153 were significantly higher on the seats than on the dashboard (*p* < 0.05, Table S15); dashboard and trunk dust were not significantly different. For example, BDE-99 had median levels of 4.7 ng/g in seat dust, compared with 1.3 ng/g in dashboard dust and 2.1 ng/g in trunk dust. These less brominated congeners are associated with Penta and OctaBDE commercial products; PentaBDE was predominantly used in polyurethane foam (PUF) in the furniture and transportation sectors (Abbasi et al., 2016; Stapleton et al., 2011), and outside of North America, predominantly in vehicle PUF (Redin et al., 2017; RPA, 2020; Secretariat of the Stockholm Convention, 2017). The higher levels of the lower brominated PBDEs in seat dust suggested the PUF seats as a potential source of PBDEs; however, we note that this difference was seen regardless of the car age, even in newer cars which should have no additive PentaBDE use. The highest levels of BDE-209 were also found in the seat dust, at median levels of 3050 ng/g, followed by the trunk at 2280 ng/g. Lower concentrations of BDE-209 on the dashboard suggested either lower use of BDE-209 in dashboard components, or debromination of BDE-209 in vehicle components due to exposure to extreme temperatures and radiation (Lagalante et al., 2011).

#### NHFRs

Hexabromobenzene (HBB), DBDPE, 1,2-bis(2,4,6-tribromophenoxy)ethane (BTBPE), ΣDBE-DBCH had similar concentrations in dust from all car parts (*p* < 0.05, Table S16). BEH-TEBP and EH-TBB had higher concentrations in seat dust compared to trunk and dashboard dust, with median concentrations in seat dust of 1150 ng/g and 19.3 ng/g, compared to concentrations of 68.0 ng/g and 2.91 ng/g, respectively, in dashboard dust. PBT, 1,3,5-tribromo-2-(2,3-dibromopropoxy)benzene (TBP-DBPE), 1,3,5-tris(2,3-dibromopropyl)-1,3,5-triazine-2,4,6(1H,3H,5H)-trione (TDBP-TAZTO), and 2,3,4,5,6-pentabromoethylbenzene (PBEB) had higher concentrations in dashboard dust; the larger set of NHFRs with slightly elevated concentrations in dashboard dust suggests that the more complex mix of materials, and in particular electronic components, may be contributing to the burden of NHFRs on dashboards. Dechlorane Plus (DDC-CO) had similar concentrations in dashboard and seat dust; however, trunk dust had the highest concentrations, at 17.3 ng/g. The ratio of DDC-CO isomers (determined as the fraction of anti-DDC-CO to ΣDDC-CO—f_anti_) did not differ between dust from different car parts and, with an average f_anti_ of 0.61, was comparable to what has been reported in environmental samples (Feo et al., 2012).

#### OPEs

TDCIPP, TCEP, and TCIPP were found in the highest concentrations on the dashboard (*p* < 0.05, Table S17), significantly higher than in dust from the trunk. TDCIPP had significantly different concentrations in dust between all locations, with dashboard (252,000 ng/g) > seats (58,900 ng/g) > trunk (1830 ng/g). TBOEP and TPhP had the highest concentrations in seat dust, with medians of 5070 ng/g and 1200 ng/g, respectively.

These large differences across car parts suggested clear differences in the use of OPEs in different vehicle polymers. Use of chlorinated OPEs was higher in dashboard components materials or electronics, while non-halogenated OPEs (TPhP and TBOEP), which also act as plasticizers (Van Der Veen & De Boer, 2012), had higher concentrations in seat dust. Although the use of non-halogenated OPEs in textiles and PUF has previously been noted (Chokwe et al., 2020; Lu et al., 2023), chlorinated OPEs, particularly TCIPP, are frequently identified as replacements for PentaBDE in foams (Secretariat of the Stockholm Convention, 2007), and have recently been reported as key FRs in vehicle seat foam (Hoehn et al., 2024). A Dutch study on car dust found levels of TDCIPP were highest on seats, suggesting its use in PUF (Brandsma et al., 2014), however, also noted higher levels of TCIPP and TCEP in dashboard dust compared with seat dust.

### Recommendations for standardization of vehicle dust sampling

In the literature on SVOCs in car dust, there are inconsistencies in the sampled vehicle areas. Our measurements suggested associations between chemical concentrations in seat and dashboard dust and potential sources of plastic additives in vehicles. Seats and dashboards are most directly linked with potential exposure through contact with dust. Thus, as the best indicator of human exposure in vehicles, we recommend a composite sample of the seats and dashboard, without the floor and trunk. In the next sections, we only consider dust from the seat and dashboard.

### Comparison with other dust concentrations

#### PFAS

Four previous studies have quantified PFAS in car dust, three of them reporting PFOA levels with medians of 1.8 to 33 ng/g, and PFOS medians of 1.3 to 15.8 ng/g (Björklund et al., 2009; Fraser et al., 2013; Harrad et al., 2019). Our levels of PFAS in car dust were generally lower than those in previous studies. For example, PFOA concentrations in UK cars had a median value of 65 ng/g (our study: 18.8 ng/g), and a median of 97.0 ng/g for PFOS (our study: 5.1 ng/g) (Goosey & Harrad, 2011). While the levels of PFOA and PFDA that we detected in cars were generally higher or similar to those measured in homes (Karásková et al., 2016; Zhang et al., 2020), PFOA and PFDA in car dust were generally lower than those in other non-residential spaces (Schildroth et al., 2022). Other PFAS were in lower or similar concentrations compared to other indoor environments (Karásková et al., 2016; Schildroth et al., 2022; Zhang et al., 2020).

#### PBDEs

Nine studies report concentrations of at least one PBDE in car dust (Ali et al., 2013; Brommer et al., 2012; Gevao et al., 2016; Harrad & Abdallah, 2011; Harrad et al., 2008, 2016a; Kalachova et al., 2012; Lagalante et al., 2009, 2011). In car dust, as well as in most indoor dust, BDE-209 typically dominates. In our study, it accounted for 99% of the total PBDEs quantified in car dust. BDE-209 medians reported in cars span a large range, from 168 to 280,000 ng/g (Fig. S8, Table S17); our median of 3260 ng/g was within this range. The highest concentrations of BDE-209 in car dust have been consistently reported in studies from the UK (medians 81,000–190,000 ng/g) (Harrad & Abdallah, 2011; Harrad et al., 2008) and USA (medians 8120–48,100 ng/g) (Lagalante et al., 2009, 2011), which may suggest higher use of BDE-209 in vehicles from these regions, and/or the impact of differing flammability regulations. This was similarly highlighted by Lagalante et al. (2009), who found evidence of higher use of BDE-209 in US and UK cars. Studies of cars conducted in Germany, the Czech Republic, Kuwait, Pakistan, and Nigeria had lower median values (168–940 ng/g) (Ali et al., 2013; Brommer et al., 2012; Gevao et al., 2016; Harrad et al., 2016a; Kalachova et al., 2012), also notably lower than in our study.

Concentrations of BDE-209 in cars were many times higher than in house dust (Fig. S8). In Czech homes, BDE-209 had a median of 139 ng/g (Venier et al., 2016), whereas our study reported a median of 3260 ng/g in cars. Typically, American homes have among the highest levels of PBDEs in settled dust due to historical past higher use of PBDEs (Abbasi et al., 2019) with medians of BDE-209 in the range of 1000–3000 ng/g (Stapleton et al., 2005; Venier et al., 2016); levels we quantified in cars even exceeded those in American homes. These elevated levels in cars strongly suggest the use of DecaBDE-containing materials. BDE-209 was present at levels exceeding other indoor environments even in the newest vehicle.

#### NHFR concentrations in indoor dust

NHFRs have received limited attention in cars, therefore comparisons across studies are difficult. BEH-TEBP, EH-TBB, and DBDPE have been most frequently reported (Ali et al., 2013; Besis et al., 2017). Other than a Brazilian study which quantified extremely high levels (BEH-TEBP median: 59,700 ng/g) (Cristale et al., 2018), the concentrations of BEH-TEBP detected in our car dust (median 734 ng/g) were higher than reported in other cars, higher than in house dust (Ali et al., 2011; Al-Omran & Harrad, 2016; Fromme et al., 2014; Shoeib et al., 2012; Stapleton et al., 2008, 2009), including in Czech and Slovak homes (Demirtepe et al., 2019; Venier et al., 2016), and also exceed those in most other indoor spaces (Ali et al., 2011; Cao et al., 2014). Higher levels have only been reported for indoor spaces in California (Allgood et al., 2017).

In contrast, EH-TBB in our car dust (median 10.1 ng/g) was comparable to what has been reported in other vehicle dust (Ali et al., 2013; Besis et al., 2017; McGrath et al., 2018) (excepting the Brazilian study with median 68,200 ng/g (Cristale et al., 2018)), as well as European house dust (medians 1–5.8 ng/g) (Ali et al., 2011; Al-Omran & Harrad, 2016), and lower than levels from Canadian, American, and Brazilian house dust (Cristale et al., 2018; Shoeib et al., 2012; Stapleton et al., 2008, 2009). The high levels of BEH-TEBP and EH-TBB in North American dust have been attributed to the use of commercial Firemaster® 550 (Stapleton et al., 2008); however, this FR was not registered in the EU (ECHA, 2022). Therefore, the high levels of BEH-TEBP in car dust without elevated levels of EH-TBB suggest the use of an alternative BEH-TEBP-containing commercial FR product in vehicles produced within the EU.

Seven previous studies have reported DBDPE in cars, with medians in the range of 65–848 ng/g in European, Pakistani, and Kuwaiti cars (Ali et al., 2013; Besis et al., 2017; Brommer et al., 2012; Harrad et al., 2008; Kalachova et al., 2012); we detected a similar range, although with a slightly lower median of 65.1 ng/g (range 13.7–7390 ng/g). Dust from Australia and Brazil had substantially higher concentrations of DBDPE in dust: 1900 ng/g in Australian car dust (McGrath et al., 2018), 1360 ng/g in Brazilian car dust (Cristale et al., 2018), which may suggest regional differences in FR use in cars, although there is insufficient data to confirm this. Concentrations of DBDPE in car dust were similar to what have been reported for home and office dust (Ali et al., 2011; Besis et al., 2017; Brown et al., 2014; Harrad et al., 2008; Kalachova et al., 2012; Stapleton et al., 2008).

#### OPFRs

Ten studies have reported concentrations of OPEs in vehicle dust, with the most information for TDCIPP, TCIPP, TCEP, and TPHP. As with other FRs, there are large ranges between studies and within the same countries. For example, two German studies with dust collection between 2010 and 2012 reported a tenfold difference in TDCIPP concentrations, with medians of 4100 ng/g vs 21,000 ng/g (Brommer et al., 2012; Harrad et al., 2016b).

TDCIPP in car dust ranged from 29 to 31,000 ng/g (Abafe & Martincigh, 2019; Abdallah & Covaci, 2014; Ali et al., 2013, 2016; Brommer & Harrad, 2015; Brommer et al., 2012; Christia et al., 2018; Harrad et al., 2016b; Velázquez-Gómez et al., 2019). The lowest concentrations of TDCIPP were found in Pakistan and Egypt (medians 29–61 ng/g) (Abdallah & Covaci, 2014; Ali et al., 2013), while the highest concentrations to date were reported in car dust from South Africa, Spain, the UK and Germany (12,770–31,000 ng/g) (Abafe & Martincigh, 2019; Brommer & Harrad, 2015; Brommer et al., 2012; Velázquez-Gómez et al., 2019). Our TDCIPP concentrations, with a median of 180,000 ng/g far exceeded other car measurements, particularly in dashboard dust, where medians exceeded 200 µg/g. Levels in the µg/g range have also been reported by Brandsma et al. (2014).

TDCIPP in cars was much higher than in house or school dust (Abafe & Martincigh, 2019; Ali et al., 2016; Brommer & Harrad, 2015; Brommer et al., 2012; Cequier et al., 2014; Christia et al., 2018; Cristale et al., 2016; Harrad et al., 2016b; Velázquez-Gómez et al., 2019), e.g., medians 120–706 ng/g in European homes (García et al., 2007; Van Den Eede et al., 2011; Cristale et al., 2016), and medians 1620–1890 ng/g in US homes (Schreder & La Guardia, 2014; Stapleton et al., 2009) and medians 2200–4000 ng/g in Japanese homes (Kanazawa et al., 2010; Mizouchi et al., 2015). These indoor values were at the lower end of the range found in cars and very low compared to our study median of 180,000 ng/g.

TCIPP concentrations in car dust had a similar trend to TDICPP; the lowest values of TCIPP (medians 100–291 ng/g) were reported in Pakistan and Egypt (Abdallah & Covaci, 2014; Ali et al., 2013). Concentrations in Saudi Arabia, Greece, Germany, Netherlands and South Africa (1605–5000 ng/g) (Abafe & Martincigh, 2019; Ali et al., 2016; Brandsma et al., 2014; Brommer et al., 2012; Christia et al., 2018; Harrad et al., 2016b) were comparable to our study median of 3340 ng/g. Substantially higher values have been reported in Australia, Kuwait, and the UK (24,000–53,000 ng/g) (Ali et al., 2013; Brommer & Harrad, 2015; Harrad et al., 2016b). TCIPP measured in our study, as well as in most other European car studies, was comparable to household and school dust (Ingerowski et al., 2001; García et al., 2007; Stapleton et al., 2009; Kanazawa et al., 2010; Van Den Eede et al., 2011; Cequier et al., 2014; Schreder & La Guardia, 2014; Mizouchi et al., 2015; Cristale et al., 2016).

TCEP has been typically reported in car dust at lower levels than TDCIPP or TCIPP (medians 75–2000 ng/g) (Abdallah & Covaci, 2014; Ali et al., 2013, 2016; Brommer & Harrad, 2015; Brommer et al., 2012; Christia et al., 2018; Fang et al., 2013; Harrad et al., 2016b; Velázquez-Gómez et al., 2019). Our measurement, with a median of 3240 ng/g, was higher than the concentrations measured in other countries, with the exception of South African cars (median: 10,200 ng/g) which generally had higher values for all OPEs (Abafe & Martincigh, 2019). As with TDCIPP, TCEP in European house dust was at the lower end of the range for car dust (median 230–1790 ng/g) (Ingerowski et al., 2001; García et al., 2007; Van Den Eede et al., 2011; Cristale et al., 2016).

We found TPhP and TBOEP at similar levels to other studies reporting car dust (Abdallah & Covaci, 2014; Ali et al., 2013, 2016; Brommer & Harrad, 2015; Brommer et al., 2012; Christia et al., 2018; Harrad et al., 2016b; Velázquez-Gómez et al., 2019), and comparable to European house dust (García et al., 2007; Van Den Eede et al., 2011; Cristale et al., 2016).

The comparison with other studies highlights that while concentrations of TCIPP, TPhP, and TBOEP in car dust were comparable to dust from other indoor environments, primarily homes, TCEP, and especially TDCIPP, were substantially higher in car dust compared to other indoor microenvironments, including in our study data. The concentrations of OPEs in cars were highly variable, which can be caused by the different use of individual OPEs by suppliers of sub-parts, or by specific car factories, but also by season of sampling, which can affect the temperature in the car (Hoehn et al., 2024) and by extension, emissions from materials. In our case, the selection of cars from just one manufacturer and the high levels of TDCIPP could indicate use of this FR by this manufacturer; however, it was not possible to find specific documentation.

### Time trends according to vehicle age

#### PFAS

As discussed above, PFAS concentrations were consistently higher on dashboards, and the highest PFAS concentrations were found in dashboard dust from 2002, 2009, and 2015 vehicles. Since 2006, companies have begun voluntarily reducing PFOA production in cooperation with the U.S. EPA, under the Stewardship Program (EPA, 2016) and in 2019 it was added to the Stockholm Convention (Secretariat of the Stockholm Convention, 2024a, b), yet no apparent shift in PFAS composition or decrease in restricted PFAS was observed in vehicles over the 25 years covered in this study. PFOA and PFOS values were found on individual parts of the car in relatively low concentrations, below 70 ng/g.

#### Flame retardants

**Fig. 4:**
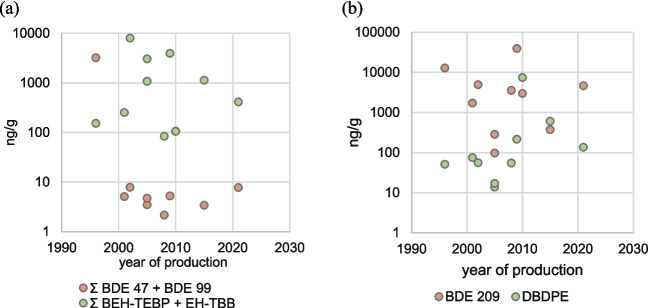
Time trends of PBDEs and associated alternative compounds in vehicle dust for **a** potential replacement of PentaBDE with Firemaster® 550 and **b** potential replacement of DecaBDE with DBDPE

A clear difference in PBDE composition was seen in the dust of the oldest car. The car from 1996 had the highest concentrations for lower brominated congeners, with BDE-47 to 154 concentrations 100–200 × higher in the car from 1996 compared to the later cars (Fig. 4a). The concentrations of BDE-47 and BDE-99 in dashboard dust from the 1996 car exceeded 3000 ng/g. BDE-47 and 99 are primary components of the formerly widely used PentaBDE product, and as the vehicle from 1996 was well within the period of unrestricted PentaBDE use (prior to 2003), we attributed the high levels in this car to direct use in the vehicle.

However, although levels in the later vehicles were substantially lower, PentaBDE components were still consistently detected at levels of ~ 3–130 ng/g. In contrast to new homes, where consistent detection of PentaBDE components is attributed to the presence of older furniture or electronics, this does not apply to post-2003 vehicles. We note that early restrictions on the use of PentaBDE included specific exemptions for the occurrence of PBDEs in partially or fully recycled materials (European Commission, 2004). BDE-99 can also be a product of debromination of BDE-209, but the inconsistent ratio between BDE-99 and BDE-209 (Table S19) suggests other sources of PentaBDE components.

Vehicle manufacturers advertise using recycled PUF in seat cushions (Basham, 2020), which may contribute to the presence of PentaBDE components in vehicle dust. The Škoda brand, the focus of our study, reports using recycled plastic for components of some models, including recycled textiles and recycled polyethylene terephthalate (PET). In the interior, some cars use recycled PET for covers and carpets (Škoda Auto a.s, 2024). Recent Škoda Octavia vehicles can be made of up to 1/3 (approx. 17 kg) of recycled plastics; new models have an even higher proportion (Jánský, 2022; Říha, 2022).

BDE-209 did not show any apparent time trend in car dust. DecaBDE, the main component of which is BDE-209, was added to the Stockholm Convention in 2017, with an exemption for use in transport sectors. The European Parliament has enacted regulations to reduce BDE-209 use in line with the Stockholm Convention, as well as thresholds in recycled materials, but also includes an exemption for the automotive and aerospace industries (European Commission, 2006). These exemptions may be why we do not observe any decrease in levels of BDE-209 in the newest vehicles. DBDPE is frequently mentioned as a replacement for BDE-209 (Vorkamp & Rigét, 2014); however, we do not see any evidence of a switch to DBDPE (Fig. 4b), and rather find that BDE-209 is still widely used in vehicles and often in orders of magnitude higher concentrations than DBDPE.

We also investigated other possible FR time trends driven by PBDE replacement. A common source of BEH-TEBP in dust is the use of Firemaster® 550 in polyurethane foams, and is cited as an alternative to PentaBDE (LANXESS, 2017; Stapleton et al., 2008; Vorkamp & Rigét, 2014), and BEH-TEBP was detected at the highest levels in seat dust (> 1000 ng/g). We examined if vehicles produced after restrictions on PentaBDE products (European Commission, 2010) may include BEH-TEBP or EH-TBB as an alternative FR, but these compounds do not show any time trend (Fig. 4a). We also considered a shift to BTBPE as a replacement for OctaBDE (Hoh and Zhu, 2005), but here too we did not see any evidence of a time trend (Fig. S9). However, since the ratio between EH-TBB and BEH-TEBP is different than that reported for the Firemaster® 550 mixture (Abdallah et al., 2019; Niu et al., 2019), it is possible that this is a different flame retardant dominated by BEH-TEBP (the median of BEH-TEBP was 734 ng/g and EH-TBB 10.4 ng/g).

Despite OPEs being considered a substitute for PBDEs (Blum et al., 2019), we do not observe any time trends. Other studies have found possible time trends for 2-ethylhexyl diphenyl phosphate (Harrad et al., 2016b) and TPhP (Tran et al., 2020), otherwise, OPE levels in dust have been linked to distance travelled rather than the age of the car for TCEP, TPhP and TBP (Tran et al., 2020), TBOEP, TDCIPP (Brommer et al., 2012), and TCIPP (Abdallah & Covaci, 2014). Harrad et al. (2016b) noted different TDCIPP levels in cars from the same year, consistent with our study, where we found a tenfold difference in TDCIPP concentrations in dust from similar vehicles.

### Limitations

This study, due to the goal of including similar cars with production in the same jurisdiction, this study only covered 10 vehicles. Consequently, not all aspects will be transferrable to other car brands, which may have more or less homogeneity in product supply chains. Additionally, while information was gathered about additional contributors to the car environments (Table S3), it was not possible to evaluate all aspects of car use that could contribute to abrasion of car interior, and affecting the relationship of vehicle dust with vehicle interior components.

## Conclusions

In our study, through quantifying levels of FRs and PFAS in 30 dust samples from 10 cars, we identified the presence of FR and PFAS levels in car dust, with selected FRs at very high levels, justifying their ongoing consideration in vehicle environments. Despite the small set of vehicles profiled, in the context of the existing body of knowledge, some key outcomes can be noted.

- The high levels of selected FRs with no decreasing time trends suggest ongoing substantive use of BDE-209, TDCIPP, TCEP and BEH-TEBP in vehicle interiors.
- There is clear heterogeneity in the composition of FRs and their levels, even when considering vehicles of similar make, model and age. This presents a challenge when attempting to generalize FR use in and exposures from cars, and suggests substantial variability in use of plastic additives in the manufacturing of vehicle parts.
- Despite the lack of enforceable flammability standards in interior materials of personal vehicles in Europe, we find clear evidence of FR use in vehicles, with levels of some FRs comparable to those in vehicles from jurisdictions with flammability standards for vehicle interior materials. This supports the contention that vehicles on the global market align with US flammability standards, even when produced in and for other markets, and suggests that contact with vehicle environments contributes to FR exposure for many populations.
- We have evidence of the impact of restrictions on PentaBDE through substantially elevated concentrations in the oldest (pre-ban) vehicle. The ongoing presence of such restricted compounds such as PentaBDE components in vehicle dust suggests continuous low-level sources of these compounds; this may be due to the increasing use of recycled polymers in vehicle interiors.

The heterogeneity and lack of information on the composition of materials in vehicles challenges our ability to generalize sources or trends. Better information on the composition and incorporation of FRs and PFAS in specific car parts is needed to understand the sources of these compounds in cars and what actions can be taken to reduce emissions/exposure without compromising safety.

## Supplementary Information

Below is the link to the electronic supplementary material.

Supplementary file1 (PDF 1288 KB)

## Data Availability

Data produced in this study are included in full in the Supplementary Information.
