# Supplementary material for: High levels of flame retardants in vehicle dust indicate ongoing use of brominated and organophosphate flame retardants in vehicle interiors
Source: Environ Monit Assess. 2025 Mar 15;197(4):396. doi: 10.1007/s10661-025-13822-z (PMC11910445; doi:10.1007/s10661-025-13822-z)
Supplement: Supplementary file 1 — Supplementary file1 (PDF 1288 KB) [file 10661_2025_13822_MOESM1_ESM.pdf]

Supporting Material for

High levels of flame retardants in vehicle dust indicate ongoing use of brominated and organophosphate flame retardants in vehicle interiors

Petra Svobodová[1], Simona Rozárka Jílková[1], Jiří Kohoutek[1], Ondřej Audy[1], Petr Šenk[1], Lisa Melymuk[1,]*

[1] RECETOX, Faculty of Science, Masaryk University, Kotlářská 2, 611 37 Brno

*** Corresponding author: Lisa Melymuk, lisa.melymuk@recetox.muni.cz**

Supporting materials containing 3 texts, 9 figures and 19 tables

Contents

List of Texts

Text S1: Extraction and cleaning process S6
Text S2: Instrumental methods S9
Text S3: QA/QC S10
References S31

List of Figures

Figure S1 Dust sampling head and quartz microfiber filter containing vehicle dust sample. S5
Figure S2 Regular sampling head with Teflon attachment and a grid for seats and trunk ... S5
Figure S3 Special sampling head used for dashboard S5
Figure S4 SRM 2528 concentrations - comparison of measured PFAS with certified values and values from scientific literature S11
Figure S5 SRM 2528 concentrations - comparison of measured PBDEs with certified values S11
Figure S6 SRM 2528 concentrations on regular (a) and logarithmic (b) scales- comparison of measured OPEs with certified values and values from scientific literature S12
Figure S7 Literature values of BDE-47 in car dust, and a comparison with settled dust levels from US and Czech homes S28
Figure S8 Literature values of BDE-209 in car dust, compared with US and Czech homes S29
Figure S9 Concentrations of octa-BDE components compared with concentration of BTBPE S30

List of Tables

Table S1 List of target compounds, abbreviations and identifiers S3
Table S2 Skoda brand cars sampled from oldest to newest along with car model and type S5
Table S3 Summary of results of questionnaire about car use and cleaning S6
Table S4 Analytical standards and suppliers S7
Table S5 Masses determined in blanks, instrumental detection limits, instrumental quantification limits and method detection limits. S13
Table S6 PFAS concentrations in individual car dust samples S14
Table S7 PFAS summary statistics S15
Table S8 PBDE concentrations in individual car dust samples S16
Table S9 PBDE summary statistics S17
Table S10 NHFR concentrations in individual car dust samples S18
Table S11 NHFR summary statistics S19
Table S12 OPE concentrations in individual car dust samples S20
Table S13 OPE summary statistics S21
Table S14 PFAS p-values and median values for separate parts of car S22
Table S15 PBDE p-values and median values for separate parts of car S24
Table S16 NHFR p-values and median values for separate parts of car S25
Table S17 OPE p-values and median values for separate parts of car S27
Table S18 Analysis of published data on PBDEs in cars in ng/g S28
Table S19 Ratio between BDE 209 and BDE 99 ..... S29

Table S1 List of target compounds, abbreviations and identifiers

	Abbreviation	Compound name	CAS RN	InChIKey	Molecular formula	Avg mass
NHFRs	TBP-AE	1,3,5-Tribromo-2-(prop-2-en-1-yloxy)benzene	3278-89-5	RZLLIOPGUFOWOD-UHFFFAOYSA-N	C9H7Br3O	370.87
	pTBX	2,3,5,6-Tetrabromo-p-xylene	23488-38-2	RXKOKVQKECXYOT-UHFFFAOYSA-N	C8H6Br4	421.75
	DBE-DBCH	1,2-Dibromo-4-(1,2-dibromoethyl)cyclohexane	3322-93-8	PQRRSJBLKOPVJV-UHFFFAOYSA-N	C8H12Br4	427.80
	TBCO	1,2,5,6-Tetrabromocyclooctane	3194-57-8	RZLXIANUDLLFHN-UHFFFAOYSA-N	C8H12Br4	427.80
	TBCT	2,3,4,5-Tetrabromo-6-chlorotoluene	39569-21-6	WMXWTOJJASZOCL-UHFFFAOYSA-N	C7H3Br4Cl	442.17
	BATE	2-Bromoallyl 2,4,6-tribromophenyl ether	99717-56-3	RLPZXGWCSHFKJI-UHFFFAOYSA-N	C9H6Br4O	449.76
	PBBZ	Benzene, pentabromo-	608-90-2	LLVVSBBXENOOQY-UHFFFAOYSA-N	C6HBr5	472.59
	PBT	Pentabromotoluene	87-83-2	OZHJEQVYCBTHJT-UHFFFAOYSA-N	C7H3Br5	486.62
	PBEB	2,3,4,5,6-Pentabromoethylbenzene	85-22-3	FIAXCDIQXHJNIX-UHFFFAOYSA-N	C8H5Br5	500.65
	DPMA	1,5-Dechlorane Plus Monoadduct	10297-21-9	LHUMZYHABWGTAF-OEWTVGTHSA-N	C13H12Cl6	380.9
	TBP-DBPE	1,3,5-Tribromo-2-(2,3-dibromopropoxy)benzene	35109-60-5	QXWYPAKUEHGJSG-UHFFFAOYSA-N	C9H7Br5O	530.67
	DBHCTD	7,8-Dibromo-1,2,3,4,11,11-hexachloro-1,4,4a,5,6,7,8,9,10,10a-decahydro-1,4-methanobenzocyclooctene	51936-55-1	XRFONNJUMOCNHA-UHFFFAOYSA-N	C13H12Br2Cl6	540.75
	EH-TBB	2-Ethylhexyl 2,3,4,5-tetrabromobenzoate	183658-27-7	HVDXCGSGEQKWGB-UHFFFAOYSA-N	C15H18Br4O2	549.92
	HBB	Hexabromobenzene	87-82-1	CAYGQBVSOZLICD-UHFFFAOYSA-N	C6Br6	551.49
	PBBA	(Pentabromophenyl)methyl acrylate	59447-55-1	GRKDVZMVHOLESV-UHFFFAOYSA-N	C10H5Br5O2	556.67
	DDC-CO	Dechlorane Plus	13560-89-9	UGQQAJOWXNCOPY-UHFFFAOYSA-N	C18H12Cl12	653.69
	BTBPE	1,2-Bis(2,4,6-tribromophenoxy)ethane	37853-59-1	YATIGPZCMOYEGE-UHFFFAOYSA-N	C14H8Br6O2	687.64
	BEH-TEBP	Bis(2-ethylhexyl) tetrabromophthalate	26040-51-7	UUEDINPOVKWVAZ-UHFFFAOYSA-N	C24H34Br4O4	706.14
	TDBP-TAZTO	1,3,5-Tris(2,3-dibromopropyl)-1,3,5-triazine-2,4,6(1H,3H,5H)-trione	52434-90-9	NZUPFZNVGSWLQC-UHFFFAOYSA-N	C12H15Br6N3O3	728.69
	DBDPE	Decabromodiphenyl ethane	84852-53-9	BZQKBFHEWDPQHD-UHFFFAOYSA-N	C14H4Br10	971.22
OPEs	TCEP	Tris(2-chloroethyl) phosphate	115-96-8	HQUQLFOMPYWACS-UHFFFAOYSA-N	C6H12Cl3O4P	285.48
	TCIPP	Tris(2-chloroisopropyl)phosphate	13674-84-5	KVMPUXDNESXNOH-UHFFFAOYSA-N	C9H18Cl3O4P	327.56
	TDCIPP	Tris(1,3-dichloro-2-propyl) phosphate	13674-87-8	ASLWPAWFJZFCKF-UHFFFAOYSA-N	C9H15Cl6O4P	430.89
	TBOEP	Tris(2-butoxyethyl) phosphate	78-51-3	WTLBZVNBAKMVDP-UHFFFAOYSA-N	C18H39O7P	398.477
	TPHP	Triphenyl phosphate	115-86-6	XZZNDPSIHUTMOC-UHFFFAOYSA-N	C18H15O4P	326.288
	CDP	Cresyl diphenyl phosphate	26444-49-5	NA	C19H17O4P	340.086
	oTMPP	Tri-o-cresyl phosphate	78-30-8	YSMRWXYRXBRSND-UHFFFAOYSA-N	C21H21O4P	368.369
	TDBPP	Tris(2,3-dibromopropyl) phosphate	126-72-7	PQYJRMFWJJONBO-UHFFFAOYSA-N	C9H15Br6O4P	697.613
	TnPP	Tripropyl phosphate	513-08-6	RXPQRKFMDQNODS-UHFFFAOYSA-N	C9H21O4P	224.237
	ip-TPP	Triphenyl phosphates isopropylated	68937-41-7	NA	C27H33O4P	452.212

	TEP	Triethyl phosphate	78-40-0	DQWPFSLDHJDLRL-UHFFFAOYSA-N	C6H15O4P	182.156
	TBP	Tributyl phosphate	126-73-8	STCOOQWBFONSKY-UHFFFAOYSA-N	C12H27O4P	266.318
	TiBP	Triisobutyl phosphate	126-71-6	HRKAMJBPFPHCSD-UHFFFAOYSA-N	C12H27O4P	266.318
PBDEs	BDE 28	2,4,4'-Tribromodiphenyl ether	41318-75-6	UPNBETHEXPIWQX-UHFFFAOYSA-N	C12H7Br3O	406.90
	BDE 66	2,3',4,4'-Tetrabromodiphenyl ether	189084-61-5	DHUMTYRHKMCVAG-UHFFFAOYSA-N	C12H6Br4O	485.80
	BDE 47	2,2',4,4'-Tetrabromodiphenyl ether	5436-43-1	XYBSIYMGXVUVGY-UHFFFAOYSA-N	C12H6Br4O	485.80
	BDE 85	2,2',3,4,4'-Pentabromodiphenyl ether	182346-21-0	DMLQSUZPTTUUDP-UHFFFAOYSA-N	C12H5Br5O	564.69
	BDE 99	2,2',4,4',5-Pentabromodiphenyl ether	60348-60-9	WHPVYXDFIXRKLN-UHFFFAOYSA-N	C12H5Br5O	564.69
	BDE 100	2,2',4,4',6-Pentabromodiphenyl ether	189084-64-8	NSKIRYMHNFTRLR-UHFFFAOYSA-N	C12H5Br5O	564.69
	BDE 153	2,2',4,4',5,5'-Hexabromodiphenyl ether	68631-49-2	RZXIRSKYBISPGF-UHFFFAOYSA-N	C12H4Br6O	643.59
	BDE 154	2,2',4,4',5,6'-Hexabromodiphenyl ether	207122-15-4	VHNPZYZQKWIWOD-UHFFFAOYSA-N	C12H4Br6O	643.59
	BDE 183	2,2',3,4,4',5',6-Heptabromodiphenyl ether	207122-16-5	ILPSCQCLBHQUEM-UHFFFAOYSA-N	C12H3Br7O	722.48
	BDE 209	2,2',3,3',4,4',5,5',6,6'-Decabromodiphenyl ether	1163-19-5	WHHGLZMJPXIBIX-UHFFFAOYSA-N	C12Br10O	959.17
PFAS	PFPA	Perfluoropropanoic acid	422-64-0	LRMSQVBRUNSOJL-UHFFFAOYSA-N	C3HF5O2	164.03
	PFBA	Perfluorobutanoic acid	375-22-4	YPJUNDFVDDCYIH-UHFFFAOYSA-N	C4HF7O2	214.04
	PFHxA	Perfluorohexanoic acid	307-24-4	PXUULQAPEKKVAH-UHFFFAOYSA-N	C6HF11O2	314.05
	PFHpA	Perfluoroheptanoic acid	375-85-9	ZWBAMYVPMDSJGQ-UHFFFAOYSA-N	C7HF13O2	364.06
	PFOA	Perfluorooctanoic acid	335-67-1	SNGREZUHAYWORS-UHFFFAOYSA-N	C8HF15O2	414.07
	PFNA	Perfluorononanoic acid	375-95-1	UZUFPBIDKMEQEQ-UHFFFAOYSA-N	C9HF17O2	464.08
	PFDA	Perfluorodecanoic acid	335-76-2	PCIUEQPBYFRTEM-UHFFFAOYSA-N	C10HF19O2	514.09
	PFUnDA	Perfluoroundecanoic acid	2058-94-8	SIDINRCMMRKXGQ-UHFFFAOYSA-N	C11HF21O2	564.09
	PFDoDA	Perfluorododecanoic acid	307-55-1	CXGONMQFMIYUJR-UHFFFAOYSA-N	C12HF23O2	614.10
	PFTrDA	Perfluorotridecanoic acid	72629-94-8	LVDGGZAZAYHXEY-UHFFFAOYSA-N	C13HF25O2	664.11
	PFTeDA	Perfluorotetradecanoic acid	376-06-7	RUDINRUXCKIXAJ-UHFFFAOYSA-N	C14HF27O2	714.12
	PFBS	Perfluorobutanesulfonic acid	375-73-5	JGTNAGYHADQMCM-UHFFFAOYSA-N	C4HF9O3S	300.09
	PFHxS	Perfluorohexanesulfonic acid	355-46-4	QZHDEAJFRJCDMF-UHFFFAOYSA-N	C6HF13O3S	400.11
	PFHpS	Perfluoroheptanesulfonic acid	375-92-8	OYGQVDSRYXATEL-UHFFFAOYSA-N	C7HF15O3S	450.12
	PFOS	Perfluorooctanesulfonic acid	1763-23-1	YFSUTJLHUFNCNZ-UHFFFAOYSA-N	C8HF17O3S	500.13
	PFDS	Perfluorodecanesulfonic acid	335-77-3	HYWZIAVPBSTISZ-UHFFFAOYSA-N	C10HF21O3S	600.14

Table S2 Skoda brand cars sampled from oldest to newest along with car model and type

Year of manufacture	Model	Car type
1996	Octavia	Combi I
2001	Fabia	6Y
2002	Fabia	Combi 6Y
2005	Fabia	Combi I
2005	Octavia	II
2008	Octavia	Scout
2009	Octavia	Combi, Octavia II
2010	Fabia	II combi
2015	Fabia	Combi
2021	Octavia	Combi V

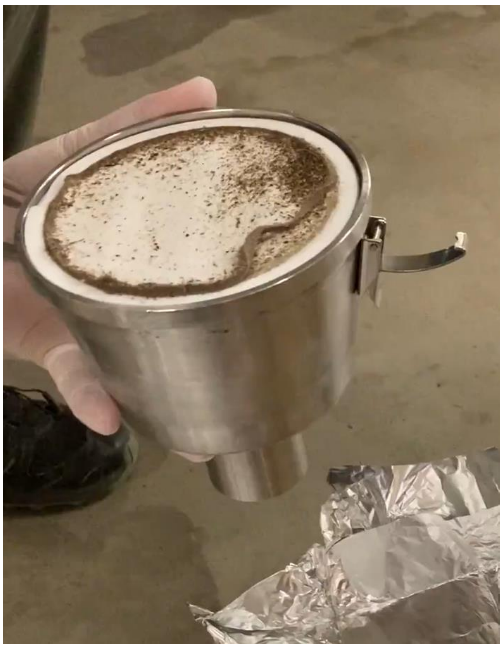

Figure S1 Dust sampling head and quartz microfiber filter containing vehicle dust sample

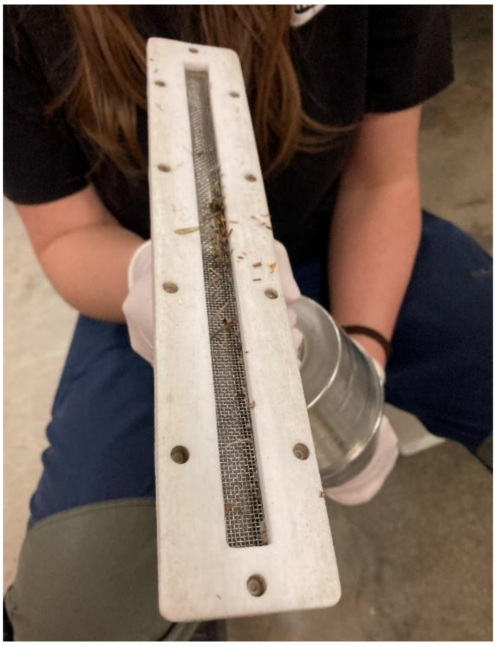

Figure S2 Regular sampling head with Teflon attachment and a grid for seats and trunk

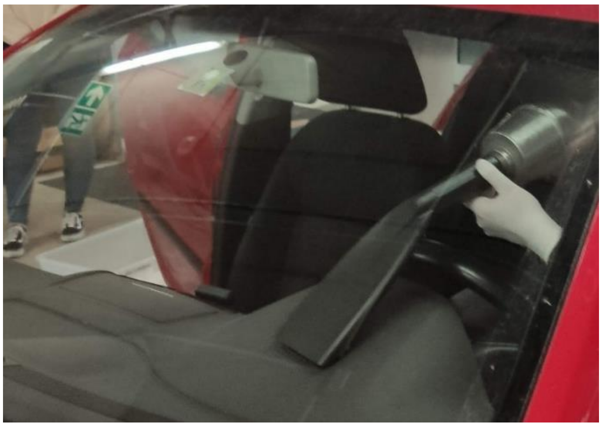

Figure S3 Special sampling head used for dashboard

Table S3 Summary of results of questionnaire about car use and cleaning

Question	Summary results
Time since last interior cleaning	1 month or less: 3 cars<br>2-6 months: 5 cars<br>1 year or more: 2 cars
Use of special interior car cleaner	Yes: 2 cars<br>No: 8 cars
Use of dashboard surface protector	Yes: 2 cars<br>No: 8 cars
Presence of air freshner	Yes, hanging: 1 car<br>Yes, on dashboard vent: 2 cars<br>No: 7 cars
Child car seats or booster seats	Yes: 4 cars<br>No: 6 cars
Other items	None: 7 cars<br>Driver heated seat cover: 1 car<br>Seat covers: 1 car<br>Truck protector: 1 car

Text S1: Extraction and cleaning process

Before extraction, the filters containing the dust samples were ground using a Ball Mill (Retsch MM 301) and kept at -4°C between processing steps.

OPEs and PFAS

The extraction of a 20% aliquot for the determination of OPEs and PFAS was carried out using methanol. A weighed 20% aliquot was transferred into glass 10 ml vials. 50 µl of recovery standard (40 µg/ml PFAS, 0,2 µg/ ml OPEs) was added to all samples. Next, 3 ml of methanol (methanol absolute, LC-MS grade, Biosolve) was added to all samples, then ultrasonic extraction was performed for 20 min, and after 20 min of sedimentation, the extract was transferred to new vials. The methanol extraction step was performed 3 times.

The extraction was followed by concentration using a flow of nitrogen and heating to 35 °C per 1 ml of extract. The samples were then purified using a nylon filter (Chromafil Xtra PA-45/13; Macherey-Nagel), and then again reduced to 0.5 ml using nitrogen flow and a temperature of 35 °C. Methanol was added to the samples to achieve a sample weight of 0.5 ml of methanol. Then 0.5 ml of Milli-Q water was added. The samples were stored in at 4 °C until analysis.

PBDEs and NHFRs

Extraction of an 80% aliquot for the determination of PBDEs and NHFRs was performed using 1:1 n-hexane-acetone (hex:acn) (n-hexane: Baker analyzed for pesticide residue analysis; J.T.Baker; acetone, Baker analyzed for pesticide residue analysis; J.T.Baker). An 80% aliquot was transferred to glass 10 ml vials. 50 µl recovery standard PBDEs, NFR (PBDE 20 pg/µl, 30 pg/µl DBDPE, 100 pg/µl BDE 209) were added to all samples. Next, 5 ml of hex:acn was added to all samples, and then ultrasonic extraction was performed for 10 min, and after 10 min of sedimentation, the extract was transferred to new vials. The hex:acn extraction step was performed 3 times, but in the second and third replicates only 3 ml of hex:acn was used for each replicate. The extraction was followed by a gentle concentration using a flow of nitrogen and heating to 32°C per 5 ml of extract.

The samples were then divided by weight into 30% and 70% fractions. Both fractions were concentrated to 0.5-1 ml before purification via column chromatography.

The 30% fraction was used for the determination of NHFRs. Purification took place in a column using 5 ml of sodium sulfate (anhydrous, granulated for organic trace analysis, Emsure, baked for 8 hours at 110°C), 5 g of activated silica gel (8 hours at 110°C) and pre-cleaned cotton wool (8 h Soxhlet with dichloromethane). The extract was added directly on sodium sulfate and gradually eluted with 10 ml of hexane. After passing through the hexane, the extract on the column was rinsed with 20 ml of dichloromethane (Baker analyzed pesticide reagent; J.T.Baker).

The 70% fraction was used for the determination of PBDEs. The purification took place in the column using 5 g of non-activated silica gel, 5 g of sulfuric silica gel (50 g of activated silica gel mixed with 22 ml of 96% H_2SO_4 -- Puriss. p.a., for determination of Hg, ACS Reagent, Reag. ISO, Reag. Ph. Eur.,Reag. USP, 95.0-97.0%, Honeywell Fluka), a 5 g of activated silica gel and pre-cleaned cotton wool. The extract was added directly on non-activated silica gel and then 30 ml of a 1:1 hexane:dichloromethane mixture was added.

50 µl of nonane (Picograde, Promochem) was added to both the 30% and 70% fractions as a final solvent. Samples were concentrated using nitrogen flow and heating at 32°C to 0.5 ml of extract. Then, the samples were transferred to conical minivials and after rinsing the previous vials, the samples were concentrated again, using nitrogen flow and heating at 32°C, down to a volume of 50 µl.

Internal standards were then added to the volume-reduced samples. 1 ng of BDEs 77 and 138 were added to the 30% fraction to quantify recoveries of NFRs. 10 µl of BDE 77 was added to the 70% fraction to quantify recoveries of PBDEs. The samples were stored at 4 ˚C until analysis.

Table S4 Analytical standards and suppliers

	Internal Standards	Concentration (µg/ml)	Spike volume (µl)	Supplier
PBDEs	13C12 PBDE 28	0.02	50	Wellington Laboratories, Canada
	13C12 PBDE 47	0.02	50	Wellington Laboratories, Canada
	13C12 PBDE 99	0.02	50	Wellington Laboratories, Canada
	13C12 PBDE 100	0.02	50	Wellington Laboratories, Canada
	13C12 PBDE 153	0.02	50	Wellington Laboratories, Canada
	13C12 PBDE 154	0.02	50	Wellington Laboratories, Canada
	13C12 PBDE 183	0.02	50	Wellington Laboratories, Canada
	13C12 PBDE 209	0.1	50	Wellington Laboratories, Canada
NFRs	13C6 PBBZ	0.02	50	Wellington Laboratories, Canada
	13C6 HBB	0.02	50	Wellington Laboratories, Canada
	13C a-DDC-CO	0.02	50	Wellington Laboratories, Canada
	13C s-DDC-CO	0.02	50	Wellington Laboratories, Canada
	13C6 BTBPE	0.02	50	Wellington Laboratories, Canada

	13C6 d17-BEH-TEBP	0.02	50	Wellington Laboratories, Canada
	13C6 d17-EH-TBB	0.02	50	Wellington Laboratories, Canada
	13C14 DBDPE	0.03	50	Wellington Laboratories, Canada

Text S2: Instrumental methods

NFRs

NFRs were analyzed using Agilent 7890A GC (Agilent Technologies, Inc., Santa Clara, CA, USA) equipped with RTX-1614 size 15 m × 0.25 mm × 0.10 µm column (Restek, Inc., France), coupled to Waters AutoSpec Premier MS (Waters Corporation, Milford, MA, USA). The GC temperature program started at 80°C (1 min hold), increased at a rate of 30°C/min to 140°C (0 min hold), followed by an increase of 4°C/min to 175°C (0 min hold), then increase at a rate of 8°C/min to 270°C (0 min hold) and finally at 15°C/min to 325°C (5 min hold). The injected sample volume was 2 µl at 250°C in pulsed splitless mode. Helium was used as a carrier gas at 1 mL/min and 1.4 mL/min after 15 min. The MS was operated in EI+ and SIM mode at the resolution of >10000.

PBDEs

PBDEs were analyzed using Agilent 7890A GC (Agilent Technologies, Inc., Santa Clara, CA, USA) equipped with RTX-1614 size 15 m × 0.25 mm × 0.10 µm column (Restek, Inc., France), coupled to Waters AutoSpec Premier MS (Waters Corporation, Milford, MA, USA). The GC temperature program started at 80°C (1 min hold), increased at a rate of 20°C/min to 250°C (0 min hold), followed by an increase of 1.5°C/min to 260°C (2 min hold) and finally by 25°C/min to 320°C (4.5 min hold). The GC/MS interface and ion source temperatures were 280 and 250°C, respectively. The injected sample volume was 2 µl at 280°C in pulsed splitless mode. Helium was used as a carrier gas at 1 mL/min and 1.4 mL/min after 15 min. The MS was operated in EI+ and SIM mode at the resolution of >10000. For BDE-209, the resolution was set to >5,000.

PFAS

PFAS were analyzed using an Agilent 1290 Series Gradient HPLC System (Agilent Technologies, Inc., Santa Clara, CA, USA) equipped with a SYNERGI 4µ Fusion Max-RP 80Ä size 100 mm × 2 mm column (Phenomenex, CA, USA) together with a Phenomenex SecurityGuard C18 size 4 × 2 mm guard column (Phenomenex, CA, USA), coupled to a QTrap 5500+ MS (SCIEX, CA, USA) with ESI source. The column was held at 30°C in a column oven. The injection volume was 10 µl. Separation was achieved using a 5 mM aqueous solution of ammonium acetate (55:45) (mobile phase A) and MeOH (mobile phase B) at a flow rate of 0.4 mL/min. The method duration was 11 minutes. Ionization was monitored in negative mode using electrospray ionization (ESI-) with the following parameters: 4500 V, a heated source at 450 °C, Ion Source Gas 1 (50 psi), Ion Source Gas 2 (30 psi), and curtain gas (15 psi). An eleven-point, calibration curve of native PFAS standards was used for quantification and results were processed in Analyst (SCIEX, CA, USA).

OPEs

Selected OPEs were analyzed using an Agilent 1290 Infinity HPLC (Agilent Technologies, Inc., Santa Clara, CA, USA) equipped with an Aquity BEH C18 size 2.1 mm × 100 mm, 1.7 µm column (Waters Corporation, Milford, MA, USA). The column was held at 30 °C in a column oven. The injection volume was 3 µl. Separation was achieved using 0.1% water solution of formic acid (mobile phase A) and 0.1% formic acid in methanol (mobile phase B) at a flow rate of 0.2 mL/min. Analyte detection was performed on an Agilent 6495 MS (Agilent Technologies, Inc., Santa Clara, CA, USA) operating in positive electrospray ionization mode

(ESI+) with the following parameters: 2700 V, a heated source at 400 °C and nitrogen as sheath gas. 13C or deuterium labelled TPHP, TnBP, TDCIPP and TnPP isotope dilution method was used for the quantification of the analytes. The linear range (MRM mode) was 0.09 – 90 µg/L, with limits of quantification from 0.01 to 0.79 µg/L for respective OPEs.

Text S3: QA/QC

Five PFAS were detected in blanks (PFDoDA, PFDA, PFNA, PFOA and PFTeDA), all at levels 0.021-0.05 ng/sample (Table S4). PFDA and PFDoDA are generally <MDL in samples due to similar levels in samples and blanks. Of the PBDEs, only BDE-209 had detectable concentrations in blanks, but the blank values were <0.4% of the median sample concentrations. Of the NHFRs, PBBZ was consistently detected in the blanks, and was excluded from further analysis. TBP-AE, BATE, α-TBCO, pTBX, TBCT and PBBA were detected in 1 or 2 dust samples at very low levels (< 2.5 ng/g), and β-TBCO, DBHCTD and DPMA were not detected in any sample. These nine FRs are not considered further in the analysis because our data do not indicate these being used in cars. Eleven NHFRs were included in the final analysis: PBT, HBB, PBEB, BEH-TEBP, EH-TBB, DBDPE, TBP-DBPE, TDBP-TAZTO, BTBPE, DDC-CO (syn- and anti-), DBE-DBCH (α-, β- and γ+δ isomers). Multiple OPEs were detected in filter blanks (Table S3). High and inconsistent blank contamination was identified for TEP and was excluded from further analysis. Three OPEs (TiBP, ip-TPP and TBP) had higher levels in the blanks, up to 59 % of the median concentration, resulting in many samples <MDL, however there remained a few samples with high detected levels of these compounds, so we retained them in the data analysis. The remainder of the OPEs were at low levels (<15% of sample mass) or not detected in the blanks. oTMPP, TDBPP, and TnPP were below detection in all samples and were not included. Nine OPEs (TCEP TCIPP, TDCIPP, TBOEP, TPhP, TiBP, CDP, ip-TPP, TBP) were included in the analysis.

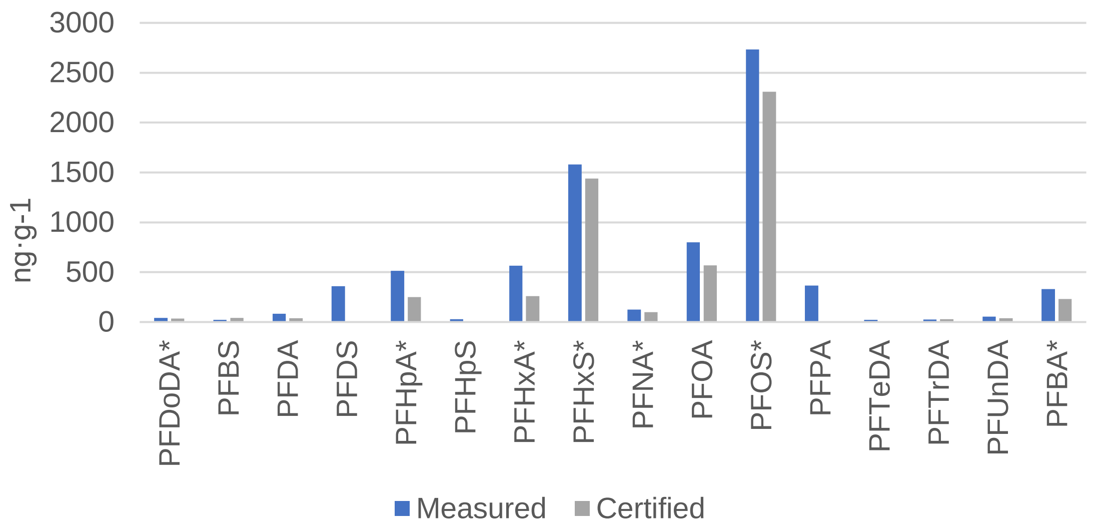

Figure S4 SRM 2528 concentrations - comparison of measured PFAS with certified values[1] and values from scientific literature [2]. Compounds with certified values are marked with the symbol [*].*

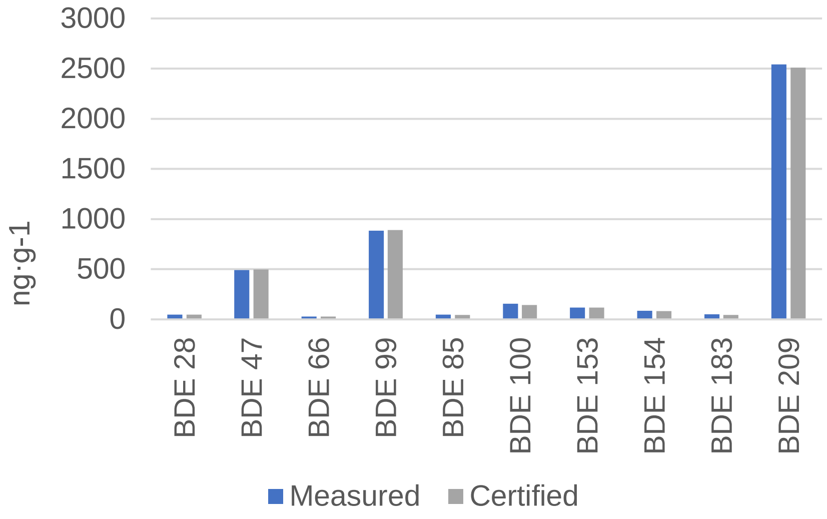

Figure S5 SRM 2528 concentrations - comparison of measured PBDEs with certified values [1].

(a)

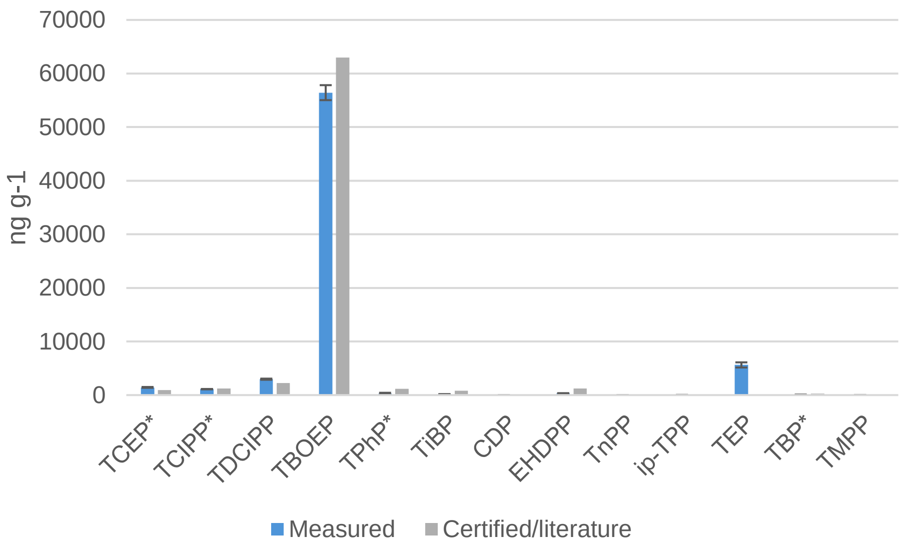

(b)

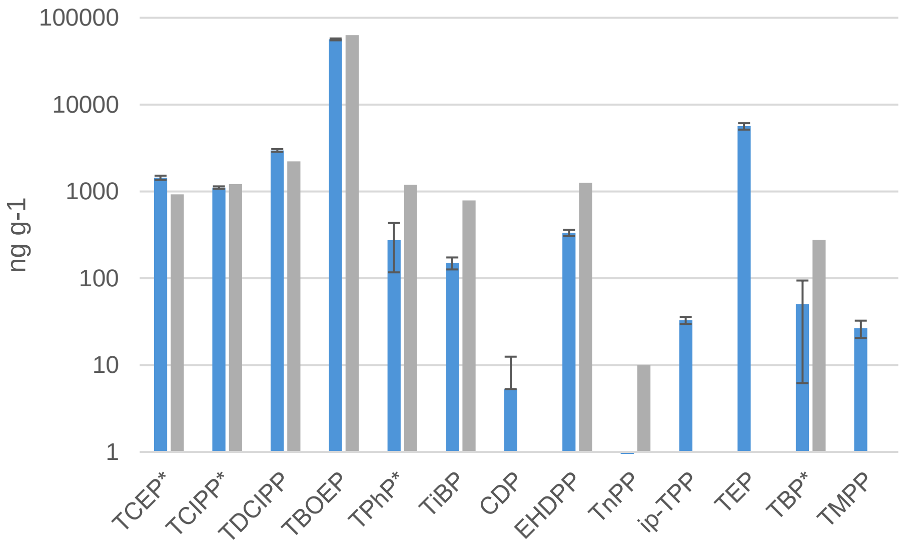

Figure S6 SRM 2528 concentrations on regular (a) and logarithmic (b) scales- comparison of measured OPEs with certified values[1] and values from scientific literature [2]. Compounds with certified values are marked with the symbol [*].*

*Table S5 Masses determined in blanks, instrumental detection limits (iLODs), instrumental quantification limits (iLOQs) and method detection limits (MDLs) calculated as average of the blanks+3*SD.*

Compound group	Compound	Average blank mass [pg/sample]	iLOD [pg/sample]	iLOQ [pg/sample]	MDL [pg/sample]
PFAS	PFDoDA	29	5	16	43
	PFBS	<iLOQ	10	40	40
	PFDA	40	4	10	53
	PFDS	<iLOQ	4	13	13
	PFHpA	<iLOQ	10	30	30
	PFHpS	<iLOQ	5	17	17
	PFHxA	<iLOQ	10	40	40
	PFHxS	<iLOQ	4	14	14
	PFNA	20	4	12	23.4
	PFOA	60	20	70	110
	PFOS	<iLOQ	30	90	90
	PFPA	<iLOQ	10	40	40
	PFTeDA	20	4	14	42.2
	PFTrDA	8	5	16	19.1
	PFUnDA		4	12	12
	PFBA		10	40	40
PBDEs	BDE 28	<iLOQ	-	1.61	1.61
	BDE 47	<iLOQ	-	1.15	1.15
	BDE 66	<iLOQ	-	1.74	1.74
	BDE 99	<iLOQ	-	2.23	2.23
	BDE 85	<iLOQ	-	2.68	2.68
	BDE 100	<iLOQ	-	1.68	1.68
	BDE 153	<iLOQ	-	5.86	5.86
	BDE 154	<iLOQ	-	3.88	3.88
	BDE 183	<iLOQ	-	8.56	8.56
	BDE 209	575	-	575	731
NHFRs	PBT	<iLOQ	-	5.64	5.64
	HBB	<iLOQ	-	4.95	4.95
	PBEB	<iLOQ	-	8.58	8.58
	BEH-TEBP	<iLOQ	-	133	133
	EH-TBB	<iLOQ	-	26.6	26.6
	DBDPE	<iLOQ	-	31.5	31.5
	TBP-DBPE	<iLOQ	-	9.32	9.32
	TDBP-TAZTO	<iLOQ	-	2.98	2.98
	BTBPE	<iLOQ	-	26.3	26.3
	a-DDC-CO	<iLOQ	-	29	29
	s-DDC-CO	<iLOQ	-	59.7	59.7
	α-DBE-DBCH	<iLOQ	-	0.246	0.246
	β-DBE-DBCH	<iLOQ	-	0.248	0.248
	γ+δ-DBE-DBCH	<iLOQ	-	0.905	0.905
OPEs	TCEP	1250	465	1550	2270
	TCIPP	16300	869	2900	25100
	TDCIPP	2840	85.8	286	7560
	TBOEP	920	25.7	86	1330
	TPHP	590	15.8	53	840
	oTMPP	<iLOQ	21	70	70
	CDP	80	9.19	31	130
	TiBP	700	55.7	186	1150
	TDBPP	<iLOQ	16.6	55	55
	TnPP	<iLOQ	4.03	13	13
	ip-TPP	110	7.28	24	240
	TEP	135000	187	622	481000
	TBP	920	17.8	59	1230

Table S6 PFAS concentrations [ng/g] in individual car dust samples (<iLOQ – below instrumental limit of quantification, <iLOD – below instrumental limit of detection, <MDL – below method detection limit)

Year	Model	Part of car	PFDoDA	PFBS	PFDA	PFDS	PFHpA	PFHpS	PFHxA	PFHxS	PFNA	PFOA	PFOS	PFPA	PFTeDA	PFTrDA	PFUnDA	PFBA
1996	Octavia	dashboard	<MDL	<iLOD	<MDL	<iLOD	17.4	<iLOD	<iLOQ	<iLOD	12.9	<MDL	<iLOD	<iLOD	<MDL	<iLOD	<iLOD	<iLOD
2001	Fabia	dashboard	<MDL	<iLOD	<MDL	<iLOD	<iLOD	<iLOD	<iLOD	<iLOD	<MDL	<MDL	<iLOD	<iLOD	<MDL	<iLOD	<iLOD	<iLOD
2002	Fabia	dashboard	<MDL	<iLOD	<MDL	<iLOD	<iLOD	<iLOD	<iLOQ	<iLOD	<MDL	<MDL	<iLOD	<iLOD	<MDL	<iLOQ	<iLOQ	<iLOD
2005	Fabia	dashboard	<MDL	<iLOQ	1.55	<iLOD	<iLOQ	<iLOD	<iLOD	<iLOQ	0.827	5.94	<iLOQ	<iLOD	1.84	<iLOQ	0.903	<iLOD
2005	Octavia	dashboard	<MDL	9.27	5.88	2.36	13.5	11.8	10.2	10.4	10.8	22.3	<iLOQ	12.4	6.67	<MDL	<iLOQ	<iLOQ
2008	Octavia	dashboard	<MDL	<iLOD	<MDL	<iLOD	<iLOD	<iLOD	<iLOD	<iLOD	<MDL	<MDL	<iLOD	<iLOD	<MDL	<iLOD	<iLOD	<iLOD
2009	Octavia	dashboard	<MDL	<iLOQ	<MDL	<iLOD	<iLOD	<iLOD	<iLOD	<iLOD	<MDL	<MDL	<iLOD	<iLOD	<MDL	<iLOD	<iLOD	<iLOD
2010	Fabia	dashboard	<iLOQ	<iLOQ	<MDL	<iLOD	<iLOD	<iLOD	<iLOD	<iLOD	<MDL	<MDL	<iLOD	<iLOD	<MDL	<iLOD	<iLOD	<iLOD
2015	Fabia	dashboard	<MDL	<iLOD	<MDL	<iLOD	26.6	<iLOD	166	<iLOD	<MDL	<MDL	<iLOD	<iLOQ	<iLOQ	<iLOQ	<iLOD	<iLOQ
2021	Octavia	dashboard	<MDL	<iLOQ	<MDL	<iLOQ	<iLOD	<iLOD	<iLOQ	<iLOD	<MDL	<MDL	<iLOD	<iLOD	7.6	<iLOQ	<iLOD	<iLOD
1996	Octavia	seats	0.886	0.512	2.13	<iLOD	0.903	<iLOD	1.38	<iLOD	0.893	3.18	<iLOD	<iLOD	0.902	0.27	0.443	<iLOD
2001	Fabia	seats	0.973	1.38	2.71	<iLOD	1.07	<iLOD	2.04	0.45	1.02	4.97	<iLOD	<iLOD	0.778	<iLOQ	0.484	2.29
2002	Fabia	seats	3.11	1.36	6.78	<iLOD	2.02	<iLOD	2.25	<iLOD	2.84	8.40	2.80	<iLOD	1.76	0.384	1.23	11.6
2005	Fabia	seats	0.894	1.08	1.78	<iLOD	0.728	<iLOD	1.42	0.200	1.06	2.99	4.51	0.35	0.818	0.201	0.706	<iLOD
2005	Octavia	seats	<MDL	9.76	4.01	4.16	10.9	7.05	11.0	8.41	6.54	13.0	9.49	10.9	4.4	<MDL	1.72	11.7
2008	Octavia	seats	<MDL	<iLOQ	1.07	<iLOD	<iLOQ	<iLOD	<iLOQ	<iLOD	1.37	2.88	6.25	1.59	<MDL	<iLOD	<iLOQ	1.47
2009	Octavia	seats	<MDL	<iLOQ	<MDL	<iLOD	<iLOQ	<iLOD	<iLOQ	<iLOD	<MDL	<MDL	<iLOQ	<iLOD	<MDL	<iLOD	<iLOQ	<iLOD
2010	Fabia	seats	<MDL	<iLOQ	1.49	5.51	<iLOQ	<iLOD	<iLOQ	<iLOD	<MDL	<MDL	5.30	<iLOD	<MDL	<iLOD	<iLOD	<iLOD
2015	Fabia	seats	<MDL	<iLOQ	<MDL	<iLOD	<iLOQ	<iLOD	6.41	<iLOD	2.95	7.52	<iLOQ	<iLOD	<MDL	<iLOD	<iLOQ	<iLOD
2021	Octavia	seats	<MDL	0.724	0.626	<iLOD	<iLOQ	<iLOD	0.999	<iLOD	0.385	2.39	2.53	0.896	0.769	<iLOD	<iLOQ	1.24
1996	Octavia	trunk	0.586	0.711	1.2	<iLOD	0.53	<iLOD	0.616	<iLOD	1.06	2.82	6.4	<iLOD	0.378	0.182	0.45	<iLOD
2001	Fabia	trunk	<MDL	<iLOQ	<MDL	<iLOD	<iLOD	<iLOD	<iLOD	<iLOQ	<MDL	<MDL	<iLOQ	<iLOD	<MDL	<iLOD	<iLOD	<iLOD
2002	Fabia	trunk	4.22	<iLOD	10.1	<iLOD	<iLOQ	<iLOD	<iLOQ	<iLOQ	2.59	18.3	<iLOQ	<iLOD	4.61	<iLOQ	1.97	<iLOD
2005	Fabia	trunk	1.16	0.986	2.64	<iLOD	1.29	0.431	1.35	0.273	1.78	4.09	6.63	4.15	0.895	0.239	0.651	<iLOD
2005	Octavia	trunk	<MDL	16.6	6.53	1.45	23.2	11.6	22.5	16.1	11.3	20.1	51.3	19.3	5.19	1.78	3.37	22.8
2008	Octavia	trunk	<MDL	<iLOQ	<MDL	<iLOD	<iLOD	<iLOD	<iLOD	<iLOD	<MDL	<MDL	<iLOD	<iLOD	<iLOQ	<iLOD	<iLOD	<iLOD
2009	Octavia	trunk	<MDL	<iLOQ	<MDL	<iLOD	<iLOD	<iLOD	<iLOQ	<iLOD	<MDL	<MDL	<iLOD	<iLOD	<MDL	<iLOD	<iLOD	<iLOQ
2010	Fabia	trunk	0.613	0.22	1.44	4.66	0.329	<iLOD	0.516	<iLOD	0.387	2.42	9.02	<iLOD	0.375	<iLOQ	0.246	0.699
2015	Fabia	trunk	<MDL	5.56	<MDL	<iLOD	<iLOD	<iLOD	6.7	<iLOD	<MDL	<MDL	<iLOD	<iLOD	<iLOQ	<iLOD	<iLOD	<iLOD
2021	Octavia	trunk	<MDL	<iLOQ	<MDL	<iLOD	<iLOD	<iLOD	<iLOQ	<iLOD	<iLOQ	<MDL	1.38	1.04	0.387	<iLOD	<iLOD	<iLOD

Table S7 PFAS summary statistics [ng/g] (DF – detection frequency, Stand. dev – standard deviation)

		PFDoDA	PFBS	PFDA	PFDS	PFHpA	PFHpS	PFHxA	PFHxS	PFNA	PFOA	PFOS	PFPA	PFTeDA	PFTrDA	PFUnDA	PFBA
Dashboard	DF	0.9	0.1	1	0.1	0.3	0.1	0.2	0.1	1	1	0	0.1	0.9	0.1	0.1	0
	Min	1.33	1.13	1.55	0.09	0.804	0.113	0.226	0.322	0.827	5.94	2.41	0.226	1.84	0.402	0.322	0.226
	Median	13.5	3.33	14.4	1.21	2.98	1.42	6.27	1.14	4.92	30.3	8.54	2.85	8.73	2.12	1.14	2.85
	Max	34.9	29.6	37.3	2.37	26.6	11.8	166	10.4	20.9	63.5	17.7	17.5	33.2	7.36	5.04	17.5
	Average	13.4	6.47	14.7	1.30	7.41	2.3	22.5	1.96	7.43	35.0	8.01	4.88	12	2.75	1.50	4.09
	Stand. dev	9.76	8.03	10	0.776	8.37	3.26	48.1	2.86	5.89	20.0	4.42	5.37	8.71	2.33	1.30	4.76
Seats	DF	1	0.6	1	0.2	0.5	0.1	0.7	0.3	1	1	0.6	0.4	1	0.4	0.5	0.5
	Min	0.264	0.512	0.626	0.004	0.345	0.004	0.633	0.004	0.385	2.39	0.027	0.009	0.711	0.043	0.084	0.009
	Median	1.11	1.22	2.02	0.043	0.985	0.053	1.47	0.103	1.06	4.07	4.08	0.327	1.04	0.188	0.486	0.823
	Max	3.11	9.76	6.78	5.51	10.9	7.05	11.0	8.41	6.54	13.0	9.49	10.9	4.40	1.28	1.72	11.7
	Average	1.50	2.01	2.51	1.01	2.02	0.771	2.87	0.954	1.89	5.34	3.97	1.49	1.53	0.288	0.615	2.92
	Stand. dev	0.896	2.62	1.68	1.94	3.01	2.09	3.13	2.49	1.75	3.23	2.67	3.18	1.08	0.344	0.485	4.41
Trunk	DF	1	0.5	1	0.2	0.4	0.2	0.5	0.2	0.9	1	0.5	0.3	0.8	0.3	0.5	0.2
	Min	0.399	0.22	0.426	0.006	0.068	0.008	0.23	0.006	0.108	1.21	0.691	0.015	0.375	0.034	0.027	0.015
	Median	1.87	1.07	3.03	0.225	0.588	0.281	1.23	0.235	1.85	6.95	6.52	0.606	1.02	0.255	0.349	0.606
	Max	6.56	16.6	10.1	4.66	23.2	11.6	22.5	16.1	11.3	20.1	51.3	19.3	6.24	1.78	3.37	22.8
	Average	2.42	3.45	3.79	0.739	3.03	1.37	3.9	2.01	2.61	9.28	9.92	2.76	2.27	0.475	0.773	2.89
	Stand. dev	1.9	4.8	3.01	1.37	6.76	3.43	6.47	4.75	3.08	7.17	14.3	5.62	2.19	0.547	1.02	6.69
Overall	DF	0.97	0.4	1	0.17	0.4	0.13	0.47	0.2	0.97	1	0.37	0.27	0.9	0.27	0.37	0.23
	Min	0.264	0.22	0.426	0.004	0.068	0.004	0.226	0.004	0.108	1.21	0.027	0.009	0.375	0.034	0.027	0.009
	Median	2.38	1.64	3.7	0.242	1.17	0.281	1.9	0.297	2.23	9.08	5.28	0.85	2.06	0.35	0.59	1.18
	Max	34.9	29.6	37.3	5.51	26.6	11.8	166	16.1	20.9	63.5	51.3	19.3	33.2	7.36	5.04	22.8
	Average	5.78	3.97	7	1.02	4.15	1.48	9.74	1.64	3.98	16.5	7.3	3.04	5.26	1.17	0.962	3.3
	Stand. dev	7.91	5.91	8.22	1.46	6.86	3.06	29.5	3.54	4.67	18.1	9.12	5.05	7.07	1.79	1.06	5.41
Seats+ dashboard	DF	0.95	0.35	1	0.15	0.4	0.1	0.45	0.2	1	1	0.3	0.25	0.95	0.25	0.3	0.25
	Min	1.11	1.11	1.66	0.0523	0.815	0.0653	0.823	0.203	0.943	4.47	3.25	0.288	1.33	0.254	0.203	0.131
	Median	7.16	2.39	8.12	0.712	2.26	0.723	3.77	0.611	3.73	18.8	5.05	1.53	5.41	1.14	0.804	2.08
	Max	18.3	15.5	19.6	3.26	14.1	9.44	86.2	9.39	11	35.2	10.7	11.7	17.5	3.78	3.13	8.96
	Average	7.47	4.24	8.6	1.16	4.71	1.54	12.7	1.46	4.66	20.2	5.99	3.18	6.75	1.52	1.06	3.51
	Stand. Dev	5.04	4.42	5.24	1.02	4.86	2.66	24.7	2.66	3.14	10.6	2.57	3.68	4.36	1.21	0.809	3.16

Table S8 PBDE concentrations [ng/g] in individual car dust samples (<iLOQ – below instrumental limit of quantification, NR – not reported

Year	Model	Part of car	BDE 28	BDE 47	BDE 66	BDE 99	BDE 85	BDE 100	BDE 153	BDE 154	BDE 183	BDE 209
1996	Octavia	dashboard	16.8	3280	61	3120	218	750	538	362	51.6	25200
2001	Fabia	dashboard	<iLOQ	0.901	<iLOQ	1.29	<iLOQ	<iLOQ	<iLOQ	<iLOQ	<iLOQ	1920
2002	Fabia	dashboard	0.787	1.58	0.458	0.948	<iLOQ	<iLOQ	<iLOQ	<iLOQ	4.18	704
2005	Fabia	dashboard	0.0293	0.213	<iLOQ	0.222	<iLOQ	0.0514	<iLOQ	<iLOQ	0.849	125
2005	Octavia	dashboard	<iLOQ	1.02	<iLOQ	2.06	<iLOQ	0.383	<iLOQ	<iLOQ	0.383	65.3
2008	Octavia	dashboard	<iLOQ	0.385	<iLOQ	0.413	<iLOQ	0.11	<iLOQ	<iLOQ	<iLOQ	147
2009	Octavia	dashboard	<iLOQ	<iLOQ	<iLOQ	1.22	<iLOQ	<iLOQ	<iLOQ	<iLOQ	9.42	8060
2010	Fabia	dashboard	0.255	12	0.133	4.96	0.164	1.2	0.285	0.195	0.736	575
2015	Fabia	dashboard	<iLOQ	0.513	<iLOQ	<iLOQ	<iLOQ	<iLOQ	<iLOQ	<iLOQ	<iLOQ	136
2021	Octavia	dashboard	NR	NR	NR	<iLOQ	<iLOQ	NR	NR	NR	NR	NR
1996	Octavia	seats	0.116	11.7	0.332	10.2	0.634	2.4	1.04	0.761	0.485	345
2001	Fabia	seats	0.0474	2.94	0.103	5.03	0.366	0.931	0.943	0.542	1.56	1500
2002	Fabia	seats	0.327	4.98	0.429	8.14	<iLOQ	1.53	1.65	1.49	20.5	9100
2005	Fabia	seats	0.0666	2.2	0.112	4.3	<iLOQ	0.684	0.878	0.457	6.61	443
2005	Octavia	seats	<iLOQ	1.97	<iLOQ	4.34	0.254	0.871	<iLOQ	0.448	0.816	129
2008	Octavia	seats	0.0415	1.56	<iLOQ	1.94	<iLOQ	0.344	1.6	0.316	13.2	6920
2009	Octavia	seats	0.0818	3.58	0.116	5.52	0.306	0.976	0.986	0.674	7.74	70300
2010	Fabia	seats	0.382	90	1.16	105	3.36	22.5	8.06	7.21	2.09	5390
2015	Fabia	seats	0.0476	2.49	<iLOQ	3.58	0.113	0.635	1.45	0.643	3.7	610
2021	Octavia	seats	0.0627	2.91	0.0795	3.52	0.278	0.697	0.519	0.33	1.08	4610
1996	Octavia	trunk	0.397	17.2	0.29	21.7	0.61	3.21	1.48	1.2	0.429	306
2001	Fabia	trunk	0.117	2.01	<iLOQ	2.56	<iLOQ	0.453	1.02	<iLOQ	5.5	3110
2002	Fabia	trunk	0.467	4.61	<iLOQ	6.67	0.258	0.914	3.92	1.15	19.8	4340
2005	Fabia	trunk	0.0978	1.01	0.16	1.51	<iLOQ	0.194	1.38	0.322	8.11	766
2005	Octavia	trunk	<iLOQ	0.633	<iLOQ	0.594	<iLOQ	0.121	0.256	0.175	1.54	113
2008	Octavia	trunk	0.198	1.52	<iLOQ	1.93	<iLOQ	0.363	1.19	<iLOQ	5.29	1850
2009	Octavia	trunk	0.0463	1.95	<iLOQ	2.25	<iLOQ	0.482	0.398	0.287	4.2	17800
2010	Fabia	trunk	0.161	6.09	0.289	7.04	0.522	1.32	1.55	0.683	4.2	5390
2015	Fabia	trunk	<iLOQ	0.784	<iLOQ	1.42	<iLOQ	<iLOQ	4.27	3.31	33.5	2700
2021	Octavia	trunk	0.023	0.743	<iLOQ	0.577	<iLOQ	<iLOQ	0.122	<iLOQ	0.163	24.9

Table S9 PBDE summary statistics [ng/g] (DF – detection frequency, Stand. dev – standard deviation)

		BDE 28	BDE 47	BDE 66	BDE 99	BDE 85	BDE 100	BDE 153	BDE 154	BDE 183	BDE 209
Dashboard	DF	0.4	0.8	0.3	0.8	0.2	0.5	0.2	0.2	0.6	0.9
	Min	0.0247	0.179	0.0127	0.198	0.023	0.0514	0.064	0.0477	0.223	65.3
	Median	0.123	0.901	0.133	1.25	0.201	0.212	0.448	0.318	0.849	575
	Max	16.8	3280	61	3120	218	750	538	362	51.6	25200
	Average	2.04	367	6.95	314	22.8	83.6	60.2	40.5	7.68	4110
	Stand. dev	5.23	1030	19.1	937	65.1	236	169	114	15.8	7850
Seats	DF	0.9	1	0.7	1	0.7	1	0.9	1	1	1
	Min	0.0142	1.56	0.0198	1.94	0.0342	0.344	0.158	0.316	0.485	129
	Median	0.0647	2.92	0.108	4.68	0.266	0.901	1.01	0.592	2.9	3050
	Max	0.382	90	1.16	105	3.36	22.5	8.06	7.21	20.5	70300
	Average	0.119	12.4	0.242	15.1	0.558	3.16	1.73	1.29	5.78	9940
	Stand. dev	0.121	26	0.332	30	0.948	6.47	2.16	2	6.22	20400
Trunk	DF	0.8	1	0.3	1	0.3	0.8	1	0.7	1	1
	Min	0.0104	0.633	0.0205	0.577	0.0268	0.0275	0.122	0.0343	0.163	24.9
	Median	0.107	1.74	0.0976	2.09	0.118	0.408	1.29	0.316	4.75	2280
	Max	0.467	17.2	0.29	21.7	0.61	3.21	4.27	3.31	33.5	17800
	Average	0.157	3.66	0.131	4.63	0.206	0.716	1.56	0.768	8.27	3640
	Stand. dev	0.149	4.84	0.106	6.09	0.194	0.915	1.36	0.931	9.95	5040
Overall	DF	0.7	0.9	0.4	0.9	0.4	0.8	0.7	0.6	0.9	0.97
	Min	0.0104	0.179	0.0127	0.198	0.023	0.0275	0.064	0.0343	0.163	24.9
	Median	0.0891	1.97	0.112	3.04	0.201	0.482	0.943	0.457	3.7	1500
	Max	16.8	3280	61	3120	218	750	538	362	51.6	70300
	Average	0.727	119	2.29	111	7.86	27.3	19.8	13.3	7.23	5960
	Stand. dev	3.05	598	11.1	560	39	137	98	65.9	11.2	13400
Seats+ dashboard	DF	0.7	0.9	0.5	0.9	0.5	0.8	0.6	0.6	0.8	0.95
	Min	0.0195	0.974	0.0499	1.18	0.0286	0.227	0.221	0.195	0.6	97.2
	Median	0.0769	1.9	0.137	3.28	0.276	0.639	0.885	0.515	2.94	3260
	Max	8.47	1650	30.7	1570	109	376	270	181	26	39200
	Average	0.978	171	3.25	165	11.7	39.2	28	18.9	6.4	7050
	Stand. dev	2.5	492	9.15	468	32.6	112	80.6	54.2	7.51	11300

Table S10 NHFR concentrations [ng/g] in individual car dust samples (<iLOQ – under instrumental limit of quantification, <iLOD – under instrumental limit of detection, <MDL – under method detection limit, NR – not reported

Year	Model	Part of car	PBT	HBB	PBEB	BEH-TEBP	EH-TBB	DBDPE[1]	TBP-DBPTE	TDBP-TAZTO	BTBPE	a-DDC-CO	s-DDC-CO	α-DBE-DBCH	β-DBE-DBCH	γ+δ-DBE-DBCH
1996	Octavia	dashboard	18.7	<iLOQ	<iLOQ	88.4	<iLOQ	93.1	<iLOQ	<iLOQ	<iLOQ	<iLOQ	3.19	9.99	7.64	<iLOQ
2001	Fabia	dashboard	2.78	<iLOQ	<iLOQ	<iLOQ	<iLOQ	88.2	<iLOQ	<iLOQ	<iLOQ	<iLOQ	<iLOQ	<iLOQ	<iLOQ	<iLOQ
2002	Fabia	dashboard	20.7	3.04	<iLOQ	1680	11.2	56.3	<iLOQ	<iLOQ	2.79	1.41	2.73	5.48	<iLOQ	<iLOQ
2005	Fabia	dashboard	0.69	<iLOQ	<iLOQ	186	8.05	6.39	<iLOQ	<iLOQ	<iLOQ	1.7	<iLOQ	1.01	0.763	<iLOQ
2005	Octavia	dashboard	1.47	<iLOQ	<iLOQ	582	2.88	16.9	<iLOQ	<iLOQ	0.582	2.11	1.84	3.44	2.17	<iLOQ
2008	Octavia	dashboard	8.96	<iLOQ	<iLOQ	<iLOQ	<iLOQ	NR	<iLOQ	<iLOQ	<iLOQ	<iLOQ	<iLOQ	<iLOQ	<iLOQ	<iLOQ
2009	Octavia	dashboard	14	<iLOQ	<iLOQ	634	<iLOQ	NR	<iLOQ	<iLOQ	<iLOQ	<iLOQ	<iLOQ	<iLOQ	<iLOQ	<iLOQ
2010	Fabia	dashboard	3.63	<iLOQ	<iLOQ	47.6	1.61	*887	<iLOQ	<iLOQ	<iLOQ	<iLOQ	<iLOQ	<iLOQ	<iLOQ	<iLOQ
2015	Fabia	dashboard	4.15	<iLOQ	<iLOQ	38.4	<iLOQ	NR	<iLOQ	<iLOQ	<iLOQ	<iLOQ	<iLOQ	<iLOQ	<iLOQ	<iLOQ
2021	Octavia	dashboard	4.57	<iLOQ	<iLOQ	44.1	<iLOQ	90.6	<iLOQ	<iLOQ	<iLOQ	9.39	4.6	<iLOQ	<iLOQ	<iLOQ
1996	Octavia	seats	0.142	0.271	0.0157	196	17.9	8.55	0.232	2.76	0.248	0.383	0.471	1.89	1.48	0.575
2001	Fabia	seats	0.376	0.55	<iLOQ	448	11	61	<iLOQ	<iLOQ	3.51	3.21	2.15	5.3	4.06	0.605
2002	Fabia	seats	0.314	0.9	0.137	14100	181	54.9	<iLOQ	11	11.4	2.41	0.769	1.34	0.957	0.372
2005	Fabia	seats	0.192	0.392	0.0173	5750	161	21.1	<iLOQ	<iLOQ	1.14	11.3	7.88	7.64	6.03	1.92
2005	Octavia	seats	<iLOQ	0.66	<iLOQ	1550	6.81	NR	<iLOQ	<iLOQ	1.27	3.07	1.99	1.02	0.916	<iLOQ
2008	Octavia	seats	1.29	19.5	<iLOQ	148	9.76	54.5	<iLOQ	<iLOQ	0.879	4.75	3.59	0.826	0.577	<iLOQ
2009	Octavia	seats	1.12	3.27	0.264	7040	199	215	<iLOQ	<iLOQ	3.99	3.31	2.58	3.28	2.69	<iLOQ
2010	Fabia	seats	8.54	1.5	0.626	146	14.6	*13900	<iLOQ	<iLOQ	0.867	4.27	3.54	<iLOQ	<iLOQ	<iLOQ
2015	Fabia	seats	1.09	0.947	<iLOQ	2130	87	603	<iLOQ	<iLOQ	<iLOQ	4.46	2	10.7	8.31	1.69
2021	Octavia	seats	0.758	0.236	<iLOQ	758	20.6	*180	<iLOQ	<iLOQ	0.589	1.01	0.814	1.56	1.33	0.56
1996	Octavia	trunk	NR	NR	NR	40.7	1.43	60.6	<iLOQ	16.8	0.513	1.76	0.91	1.46	1.15	<iLOQ
2001	Fabia	trunk	0.761	4.54	<iLOQ	88	2.39	35.8	<iLOQ	<iLOQ	4.63	14.6	11.7	<iLOQ	<iLOQ	<iLOQ
2002	Fabia	trunk	0.572	1.8	<iLOQ	175	10.6	190	<iLOQ	47.2	34.5	4.99	3.25	4.94	3.29	<iLOQ
2005	Fabia	trunk	0.176	0.246	0.0546	181	9.34	47.7	1.65	<iLOQ	8.96	107	79	0.663	0.502	<iLOQ
2005	Octavia	trunk	<iLOQ	<iLOQ	<iLOQ	30.6	<iLOQ	18.5	<iLOQ	<iLOQ	1.37	34.8	3.62	3.55	2.4	<iLOQ
2008	Octavia	trunk	0.666	1.04	<iLOQ	175	10.4	60.9	<iLOQ	<iLOQ	6.78	58.4	43.2	<iLOQ	<iLOQ	<iLOQ
2009	Octavia	trunk	0.561	0.78	<iLOQ	128	4.9	20	<iLOQ	<iLOQ	<iLOQ	3.9	3.28	1.07	<iLOQ	<iLOQ
2010	Fabia	trunk	1.06	1.01	0.133	323	11.7	428	4.11	10.3	5.6	57.4	45.2	1.77	1.28	0.515
2015	Fabia	trunk	7.14	2.32	1.07	44.9	1.89	*23100	<iLOQ	<iLOQ	3	4.97	<iLOQ	<iLOQ	<iLOQ	<iLOQ
2021	Octavia	trunk	0.195	0.121	<iLOQ	11.4	<iLOQ	8.6	<iLOQ	<iLOQ	0.289	0.805	0.557	0.369	0.324	<iLOQ

[1] compounds marked with an asterisk [*] are those for which internal calibration could be used to quantify DBDPEs. Other samples were quantified based on external calibration, as described in QA/QC section.

Table S11 NHFR summary statistics [ng/g] (DF – detection frequency, Avg – Average; Stand. dev – standard deviation)

		PBT	HBB	PBEB	BEH-TEBP	EH-TBB	DBDPE	TBP-DBPTE	TDBP-TAZTO	BTBPE	a-DDC-CO	s-DDC-CO	α-DBE-DBCH	β-DBE-DBCH	γ+δ-DBE-DBCH
Dashboard	DF	1	0.1	0	0.8	0.4	0.9	0	0	0.2	0.4	0.4	0.4	0.3	0
	Min	0.69	0.013	0.0209	8.57	0.804	6.39	0.184	0.0589	0.191	0.374	0.757	0.0167	0.0168	0.0179
	Median	4.36	0.325	0.468	68	2.91	88.2	1.75	0.558	1.57	2.58	3.89	0.101	0.07	0.17
	Max	20.7	3.04	2.59	1680	11.2	887	4.85	1.55	9.32	9.39	11.7	9.99	7.64	0.471
	Avg	7.97	0.907	0.644	334	4.5	177	1.86	0.595	2.63	3.29	4.8	2.03	1.1	0.181
	Stand. dev	6.95	1.06	0.713	498	3.47	292	1.32	0.421	2.62	2.69	3.22	3.19	2.27	0.128
Seats	DF	0.9	1	0.5	1	1	0.8	0.1	0.2	0.9	1	1	0.9	0.9	0.6
	Min	0.142	0.236	0.0157	146	6.81	8.55	0.0278	0.0089	0.248	0.383	0.471	0.0043	0.0043	0.0104
	Median	0.567	0.78	0.0658	1150	19.3	61	0.134	0.0646	1.01	3.26	2.07	1.72	1.4	0.466
	Max	8.54	19.5	0.626	14100	199	13900	0.329	11	11.4	11.3	7.88	10.7	8.31	1.92
	Avg	1.41	2.82	0.164	3230	70.8	1680	0.16	1.42	2.42	3.82	2.58	3.36	2.63	0.581
	Stand. dev	2.41	5.63	0.194	4310	75.3	4320	0.106	3.31	3.24	2.83	2.04	3.3	2.56	0.658
Trunk	DF	0.8	0.8	0.3	1	0.8	0.9	0.2	0.3	0.9	1	0.9	0.7	0.6	0.1
	Min	0.176	0.121	0.0133	11.4	0.547	8.6	0.0118	0.0136	0.289	0.805	0.557	0.0047	0.0048	0.0011
	Median	0.572	1.01	0.133	108	3.65	54.2	0.436	0.146	3.82	9.78	3.45	0.869	0.413	0.0361
	Max	7.14	4.54	1.07	323	11.7	23100	4.11	47.2	34.5	107	79	4.94	3.29	0.515
	Avg	1.28	1.36	0.261	120	5.56	2400	0.862	7.51	6.65	28.8	19.1	1.39	0.9	0.0796
	Stand. dev	2.09	1.31	0.328	91.5	4.21	6910	1.17	14.3	9.7	33.5	25.8	1.57	1.09	0.147
Overall	DF	0.90	0.63	0.27	0.93	0.73	0.87	0.1	0.2	0.67	0.80	0.77	0.67	0.60	0.23
	Min	0.142	0.013	0.0133	8.57	0.547	6.39	0.0118	0.0089	0.191	0.374	0.471	0.0043	0.0043	0.0011
	Median	1.06	0.662	0.137	175	8.5	60.8	0.375	0.18	1.32	3.78	3.22	1.05	0.67	0.0776
	Max	20.7	19.5	2.59	14100	199	23100	4.85	47.2	34.5	107	79	10.7	8.31	1.92
	Avg	3.63	1.71	0.36	1230	27	1550	0.961	3.18	3.9	12	8.84	2.26	1.54	0.28
	Stand. dev	5.47	3.54	0.516	2880	53.5	5070	1.24	9.04	6.4	22.8	16.7	2.92	2.22	0.451
Seats+dashb	DF	0.95	0.55	0.25	0.9	0.7	0.85	0.05	0.1	0.55	0.7	0.7	0.65	0.6	0.3
	Min	0.441	0.203	0.0191	78.3	4.84	13.7	0.106	0.0339	0.666	0.473	1.75	0.0105	0.0106	0.0385
	Median	3.89	0.701	0.327	734	10.4	65.1	0.916	0.293	1.16	3.77	3.68	2.46	1.48	0.317
	Max	10.5	9.86	1.43	7900	104	7390	2.55	5.91	7.1	6.48	7.16	5.94	4.56	0.982
	Avg	4.69	1.87	0.404	1780	37.7	861	1.01	1.01	2.52	3.55	3.69	2.69	1.87	0.381
	Stand. dev	3.42	2.79	0.367	2380	39.2	2180	0.678	1.71	2.36	1.68	1.77	3.18	1.95	1.56

Table S12 OPE concentrations [ng/g] in individual car dust samples (<iLOQ – under instrumental limit of quantification, <iLOD – under instrumental limit of detection, <MDL – under method detection limit,)

Year	Model	Part of car	TCEP	TCIPP	TDCIPP	TBOEP	TPhP	TiBP	CDP	ip-TPP	TBP
1996	Octavia	dashboard	21800	28200	464000	939	17300	<iLOD	<MDL	81.6	<MDL
2001	Fabia	dashboard	4120	<MDL	143000	482	3050	<iLOQ	<MDL	25.1	<MDL
2002	Fabia	dashboard	54600	19800	1430000	772	<MDL	<iLOD	811	58.5	<iLOQ
2005	Fabia	dashboard	1050	1740	29000	167	70.6	<iLOD	<MDL	22.1	<iLOQ
2005	Octavia	dashboard	766	1660	30600	861	<MDL	<iLOD	<MDL	<iLOQ	<iLOQ
2008	Octavia	dashboard	11600	<MDL	473000	1730	<MDL	<iLOD	<MDL	<iLOQ	<iLOQ
2009	Octavia	dashboard	24900	<MDL	963000	1210	<MDL	<iLOD	<MDL	<iLOD	<iLOQ
2010	Fabia	dashboard	3590	2430	126000	970	306	<iLOD	<MDL	205	<MDL
2015	Fabia	dashboard	5710	<MDL	261000	644	1090	<iLOD	218	<iLOD	<iLOQ
2021	Octavia	dashboard	6880	20700	244000	277	332	<iLOD	<MDL	13.8	<MDL
1996	Octavia	seats	<iLOD	7360	946	474	3590	0.366	13.4	82.4	136
2001	Fabia	seats	151	1480	2340	6850	23800	3.21	22	218	<MDL
2002	Fabia	seats	888	1750	37700	3290	813	<MDL	374	86.3	<MDL
2005	Fabia	seats	205	2940	1040	677	503	<MDL	14.7	46.1	<MDL
2005	Octavia	seats	343	795	280000	19100	1150	<iLOD	62.6	64.7	<MDL
2008	Octavia	seats	337	776	80100	7310	622	<iLOD	22.3	32.3	<MDL
2009	Octavia	seats	262	3050	85800	8510	1260	<iLOD	34.1	108	<MDL
2010	Fabia	seats	962	4040	88200	740	2130	<iLOD	<MDL	2690	<MDL
2015	Fabia	seats	<MDL	1770	148000	8570	23800	<iLOD	28	132	<MDL
2021	Octavia	seats	274	3640	23200	609	739	<iLOQ	9.89	28.3	<MDL
1996	Octavia	trunk	610	18000	477	216	2970	0.901	201	22.2	<MDL
2001	Fabia	trunk	314	<MDL	13300	1780	1440	<MDL	123	1280	<MDL
2002	Fabia	trunk	1130	4560	7020	1260	2130	<iLOD	235	273	<MDL
2005	Fabia	trunk	374	1680	620	2070	962	2.57	28.5	297	<MDL
2005	Octavia	trunk	<iLOQ	1660	2760	1110	1110	<iLOD	464	18.5	<MDL
2008	Octavia	trunk	749	886	1450	515	627	<iLOD	77.4	90.3	<MDL
2009	Octavia	trunk	<iLOQ	1520	2830	4670	697	<iLOD	<MDL	122	<MDL
2010	Fabia	trunk	186	1660	356	292	241	<iLOD	15.4	41.9	<MDL
2015	Fabia	trunk	<iLOQ	5680	2210	206	1750	<iLOD	51	115	<MDL
2021	Octavia	trunk	396	2350	248	157	263	<iLOD	156	26.6	<MDL

Table S13 OPE summary statistics [ng/g] (DF – detection frequency, Avg – Average; Stand. dev – standard deviation)

		TCEP	TCIPP	TDCIPP	TBOEP	TPhP	TiBP	CDP	ip-TPP	TBP
Dashboard	DF	1	1	1	1	1	1	0.8	0.6	1
	Min	766	1660	29000	167	70.6	13.8	3.22	0.712	11.6
	Median	6300	4620	252000	817	347	139	18	4.2	79.2
	Max	54600	28200	1430000	1730	17300	811	205	806	304
	Average	13500	9500	416000	805	2350	205	42.6	88.8	111
	Stand. dev	15800	9220	429000	434	5060	224	59.6	239	82.7
Seats	DF	0.9	1	1	1	1	1	1	1	1
	Min	0.417	776	946	474	503	9.35	28.3	0.272	7.48
	Median	268	2360	58900	5070	1200	22.1	84.4	5.74	14.1
	Max	962	7360	280000	19100	23800	374	2690	802	61.6
	Average	350	2760	74800	5610	5840	59.1	349	122	21
	Stand. dev	306	1870	82600	5560	9020	106	784	242	16
Trunk	DF	1	1	1	1	1	1	1	1	1
	Min	80.9	886	248	157	241	15.4	18.5	0.0525	9.23
	Median	344	1670	1830	814	1040	100	102	5.34	26.7
	Max	1130	18000	13300	4670	2970	464	1280	66.9	365
	Average	408	3940	3130	1230	1220	138	228	15.4	95.1
	Stand. dev	318	4910	3900	1320	824	130	362	21.2	116
Overall	DF	0.97	1	1	1	1	1	0.9	0.8	1
	Min	0.417	776	248	157	70.6	9.35	3.22	0.0525	7.48
	Median	503	2500	29800	900	887	59.1	61.6	5.04	32
	Max	54600	28200	1430000	19100	23800	811	2690	806	365
	Average	4750	5400	165000	2550	3140	134	207	75.4	75.6
	Stand. dev	11000	6790	310000	3960	6300	172	515	202	91.5
Seats+ dashboard	DF	0.95	1	1	1	1	1	0.9	0.8	1
	Min	554	1230	15000	422	287	14.3	19.7	0.558	11.9
	Median	3240	3340	180000	2850	798	79.9	62.3	5.79	51.5
	Max	27700	17800	732000	9990	13400	593	1450	804	167
	Average	6920	6130	245000	3210	4090	132	196	105	65.8
	Stand. dev	7960	5280	210000	2850	5290	162	419	238	42.3

Table S14 PFAS p-values and median values for separate parts of car

Compound	Parts	U	Z	p-value (Exact Prob>|U|)	Asymp. Prob>|U|	significantly different?	Dashboard [ng/g]	Seats [ng/g]	Trunk [ng/g]
PFDoDA	dashboard vs seat	92	3.13711	7.25E-04	0.00171	yes			
PFDoDA	seats vs trunk	39	-0.79373	0.43587	0.42736	**no**	13.5	1.11	1.86
PFDoDA	trunk vs dashboard	88	2.83473	0.00288	0.00459	yes			
PFBS	dashboard vs seat	86	2.68355	0.0052	0.00728	yes			
PFBS	seats vs trunk	52	0.11339	0.9118	0.90972	**no**	3.33	1.22	1.07
PFBS	trunk vs dashboard	74	1.77643	0.07526	0.07566	**no**			
PFDA	dashboard vs seat	91	3.06151	0.00105	0.0022	yes			
PFDA	seats vs trunk	42	-0.56695	0.57874	0.57075	**no**	14.4	2.02	3.03
PFDA	trunk vs dashboard	87	2.75914	0.00389	0.0058	yes			
PFDS	dashboard vs seat	78	2.0788	0.03546	0.03764	yes			
PFDS	seats vs trunk	39	-0.79373	0.43587	0.42736	**no**	1.21	0.0425	0.225
PFDS	trunk vs dashboard	77	2.00321	0.04326	0.04515	yes			
PFHpA	dashboard vs seat	79	2.1544	0.02881	0.03121	yes			
PFHpA	seats vs trunk	62	0.86932	0.39305	0.38467	**no**	2.98	0.985	0.588
PFHpA	trunk vs dashboard	83	2.45677	0.0115	0.01402	yes			
PFHpS	dashboard vs seat	89	2.91033	0.00209	0.00361	yes			
PFHpS	seats vs trunk	33	-1.24728	0.21756	0.21229	**no**	1.42	0.0532	0.281
PFHpS	trunk vs dashboard	83	2.45677	0.0115	0.01402	yes			
PFHxA	dashboard vs seat	71	1.54965	0.12301	0.12122	**no**			
PFHxA	seats vs trunk	59	0.64254	0.52885	0.52052	**no**	6.27	1.47	1.23
PFHxA	trunk vs dashboard	70	1.47406	0.14314	0.14047	**no**			
PFHxS	dashboard vs seat	89	2.91033	0.00209	0.00361	yes			
PFHxS	seats vs trunk	39	-0.79373	0.43587	0.42736	**no**	1.14	0.103	0.235
PFHxS	trunk vs dashboard	78	2.0788	0.03546	0.03764	yes			
PFNA	dashboard vs seat	84	2.53236	0.00893	0.01133	yes			
PFNA	seats vs trunk	45	-0.34017	0.73936	0.73373	**no**	4.92	1.06	1.85
PFNA	trunk vs dashboard	82	2.38118	0.01469	0.01726	yes			
PFOA	dashboard vs seat	97	3.51507	7.58E-05	4.40E-04	yes			
PFOA	seats vs trunk	39	-0.79373	0.43587	0.42736	**no**	30.3	4.07	6.95
PFOA	trunk vs dashboard	89	2.91033	0.00209	0.00361	yes			
PFOS	dashboard vs seat	74	1.77643	0.07526	0.07566	**no**			
PFOS	seats vs trunk	36	-1.0205	0.315	0.30749	**no**	8.54	4.08	6.52
PFOS	trunk vs dashboard	63	0.94491	0.35268	0.3447	**no**			
PFPA	dashboard vs seat	84	2.53236	0.00893	0.01133	yes			
PFPA	seats vs trunk	38	-0.86932	0.39305	0.38467	**no**	2.85	0.327	0.606
PFPA	trunk vs dashboard	75	1.85203	0.06301	0.06402	**no**			
PFTeDA	dashboard vs seat	98	3.59066	4.33E-05	3.30E-04	yes			
PFTeDA	seats vs trunk	52	0.11339	0.9118	0.90972	**no**	8.73	1.04	1.02
PFTeDA	trunk vs dashboard	93	3.2127	4.87E-04	0.00131	yes			
PFTrDA	dashboard vs seat	97	3.51507	7.58E-05	4.40E-04	yes	2.12	0.188	0.255

PFTrDA	seats vs trunk	41	-0.64254	0.52885	0.52052	**No**			
PFTrDA	trunk vs dashboard	90	2.98592	0.0015	0.00283	Yes			
PFUnDA	dashboard vs seat	78	2.0788	0.03546	0.03764	yes			
PFUnDA	seats vs trunk	55	0.34017	0.73936	0.73373	**no**	1.14	0.486	0.349
PFUnDA	trunk vs dashboard	79	2.1544	0.02881	0.03121	yes			
PFBA	dashboard vs seat	71	1.54965	0.12301	0.12122	**no**			
PFBA	seats vs trunk	54	0.26458	0.79594	0.79134	**no**	2.85	0.823	0.606
PFBA	trunk vs dashboard	80	2.22999	0.02323	0.02575	yes			

Table S15 PBDE p-values and median values for separate parts of car

Compound	Parts	U	Z	p-value (Exact Prob>|U|)	Asymp. Prob>|U|	significanly differrent	Dashboard [pg/g]	Seats [pg/g]	Trunk [pg/g]
BDE 28	dashboard vs seat	54	0.69402	4.97E-01	0.48767	no			
BDE 28	seats vs trunk	42	-0.56695	0.57874	0.57075	no	123	65	107
BDE 28	trunk vs dashboard	50	0.36742	0.7197	0.7133	no			
BDE 47	dashboard vs seat	20	-2.00042	0.04347	0.04546	yes			
BDE 47	seats vs trunk	73	1.70084	0.08921	0.08897	no	901	2920	1740
BDE 47	trunk vs dashboard	31	-1.10227	0.27751	0.27034	no			
BDE 66	dashboard vs seat	51	0.44907	0.66072	0.65338	no			
BDE 66	seats vs trunk	56	0.41576	0.68421	0.67758	no	133	108	97.6
BDE 66	trunk vs dashboard	59	1.10227	0.27751	0.27034	no			
BDE 99	dashboard vs seat	23	-2.00321	0.04326	0.04515	yes			
BDE 99	seats vs trunk	75	1.85203	0.06301	0.06402	no	1250	4680	2090
BDE 99	trunk vs dashboard	35	-1.0961	0.27986	0.27304	no			
BDE 85	dashboard vs seat	50	0	1	1	no			
BDE 85	seats vs trunk	66	1.17169	0.24745	0.24132	no	201	266	118
BDE 85	trunk vs dashboard	64	1.0205	0.315	0.30749	no			
BDE 100	dashboard vs seat	18	-2.16372	0.02793	0.03049	yes			
BDE 100	seats vs trunk	76	1.92762	0.05243	0.0539	no	212	901	408
BDE 100	trunk vs dashboard	39	-0.44907	0.66072	0.65338	no			
BDE 153	dashboard vs seat	19	-2.08207	0.03499	0.03734	yes			
BDE 153	seats vs trunk	49	-0.0378	0.97051	0.96985	no	448	1010	1290
BDE 153	trunk vs dashboard	26	-1.51052	0.1333	0.13091	no			
BDE 154	dashboard vs seat	26	-1.51052	0.1333	0.13091	no			
BDE 154	seats vs trunk	67	1.24728	0.21756	0.21229	no	318	592	316
BDE 154	trunk vs dashboard	39	-0.44907	0.66072	0.65338	no			
BDE 183	dashboard vs seat	31	-1.10227	0.27751	0.27034	no			
BDE 183	seats vs trunk	46	-0.26458	0.79594	0.79134	no	849	2900	4750
BDE 183	trunk vs dashboard	31	-1.10227	0.27751	0.27034	no			
BDE 209	dashboard vs seat	31	-1.10227	2.78E-01	2.70E-01	no			
BDE 209	seats vs trunk	59	0.64254	0.52885	0.52052	no	575000	3050000	2280000
BDE 209	trunk vs dashboard	37	-0.61237	0.54896	0.54029	no			

Table S16 NHFR p-values and median values for separate parts of car

Compound	Parts	U	Z	p-value (Exact Prob>|U|)	Asymp. Prob>|U|	significanly different	Dashboard [pg/g]	Seats [pg/g]	Trunk [pg/g]
PBT	dashboard vs seat	90	2.98592	1.50E-03	0.00283	yes			
PBT	seats vs trunk	47	0.12247	0.90483	0.90252	**no**	4360	567	572
PBT	trunk vs dashboard	82	2.98021	0.00145	0.00288	yes			
HBB	dashboard vs seat	32	-1.32288	0.19032	0.18588	**no**			
HBB	seats vs trunk	43	-0.12247	0.90483	0.90252	**no**	325	780	1010
HBB	trunk vs dashboard	30	-1.18392	0.24281	0.23644	**no**			
PBEB	dashboard vs seat	80	2.22999	0.02323	0.02575	yes			
PBEB	seats vs trunk	36	-0.69402	0.4967	0.48767	**no**	468	65.8	133
PBEB	trunk vs dashboard	63	1.42887	0.1564	0.15304	**no**			
BEH-TEBP	dashboard vs seat	16	-2.53236	0.00893	0.01133	yes			
BEH-TEBP	seats vs trunk	91	3.06151	0.00105	0.0022	yes	68000	1150000	108000
BEH-TEBP	trunk vs dashboard	55	0.34017	0.73936	0.73373	**no**			
EHEH-TBB	dashboard vs seat	5	-3.36388	2.06E-04	7.69E-04	yes			
EHEH-TBB	seats vs trunk	92	3.13711	7.25E-04	0.00171	yes	2910	19300	3650
EHEH-TBB	trunk vs dashboard	45	-0.34017	0.73936	0.73373	**no**			
DBDPE	dashboard vs seat	28	-0.31755	0.75769	0.75082	**no**			
DBDPE	seats vs trunk	53	0.61237	0.54896	0.54029	**no**	88200	61000	54200
DBDPE	trunk vs dashboard	36	0.0488	0.96226	0.96108	**no**			
TBP-DBPTE	dashboard vs seat	96	3.43948	1.30E-04	5.83E-04	yes			
TBP-DBPTE	seats vs trunk	24	-1.92762	0.05243	0.0539	**no**	1750	134	436
TBP-DBPTE	trunk vs dashboard	79	2.1544	0.02881	0.03121	yes			
TDBP-TAZTO[1]	dashboard vs seat	77	2.00321	0.04326	0.04515	yes			
TDBP-TAZTO[1]	seats vs trunk	33	-1.24728	0.21756	0.21229	**no**	558	64.6	146
TDBP-TAZTO[1]	trunk vs dashboard	64	1.0205	0.315	0.30749	**no**			
BTBPE	dashboard vs seat	56	0.41576	0.68421	0.67758	**no**			
BTBPE	seats vs trunk	33	-1.24728	0.21756	0.21229	**no**	1570	1010	3820
BTBPE	trunk vs dashboard	37	-0.94491	0.35268	0.3447	**no**			
a-DDC-CO	dashboard vs seat	40	-0.71813	4.81E-01	4.73E-01	**no**			
a-DDC-CO	seats vs trunk	23	-2.00321	0.04326	0.04515	yes	2580	3260	9780
a-DDC-CO	trunk vs dashboard	21	-2.1544	0.02881	0.03121	yes			
s-DDC-CO	dashboard vs seat	71	1.54965	0.12301	0.12122	**no**	3890	2070	3450
s-DDC-CO	seats vs trunk	32	-1.32288	0.19032	0.18588	**no**			

s-DDC-CO	trunk vs dashboard	45	-0.34017	0.73936	0.73373	**no**			
α-DBE-DBCH	dashboard vs seat	32	-1.32288	0.19032	0.18588	**no**			
α-DBE-DBCH	seats vs trunk	69	1.39847	0.16549	0.16197	**no**	101	1720	869
α-DBE-DBCH	trunk vs dashboard	50	0	1	1	**no**			
β-DBE-DBCH	dashboard vs seat	24	-1.92762	5.24E-02	5.39E-02	**no**			
β-DBE-DBCH	seats vs trunk	73	1.70084	0.08921	0.08897	**no**	70.0	1400	413
β-DBE-DBCH	trunk vs dashboard	51	0.0378	9.71E-01	0.96985	**no**			
γ+δ-DBE-DBCH	dashboard vs seat	39	-0.79373	4.36E-01	4.27E-01	**no**			
γ+δ-DBE-DBCH	seats vs trunk	74	1.77643	0.07526	0.07566	**no**	170	466	36.1
γ+δ-DBE-DBCH	trunk vs dashboard	83	2.45677	0.0115	0.01402	yes			

Table S17 OPE p-values and median values for separate parts of car

Compound	Parts	U	Z	p-value (Exact Prob>|U|)	Asymp. Prob>|U|	significanly different	Dashboard [ng/g]	Seats [ng/g]	Trunk [ng/g]
TCEP	dashboard vs seat	98	3.59066	4.33E-05	3.30E-04	yes			
TCEP	seats vs trunk	43	-0.49135	0.63053	0.62318	**no**	6300	268	344
TCEP	trunk vs dashboard	98	3.59066	4.33E-05	3.30E-04	yes			
TCIPP	dashboard vs seat	74	1.77643	0.07526	0.07566	**no**			
TCIPP	seats vs trunk	51	0.0378	0.97051	0.96985	**no**	4620	2360	1670
TCIPP	trunk vs dashboard	77	2.00321	0.04326	0.04515	yes			
TDCIPP	dashboard vs seat	82	2.38118	1.47E-02	0.01726	yes			
TDCIPP	seats vs trunk	84	2.53236	0.00893	0.01133	yes	252000	58900	1830
TDCIPP	trunk vs dashboard	100	3.74185	1.08E-05	1.83E-04	yes			
TBOEP	dashboard vs seat	27	-1.70084	8.92E-02	8.90E-02	**no**			
TBOEP	seats vs trunk	78	2.0788	0.03546	0.03764	yes	817	5070	814
TBOEP	trunk vs dashboard	46	-0.26458	0.79594	0.79134	**no**			
TPhP	dashboard vs seat	21	-2.1544	0.02881	0.03121	yes			
TPhP	seats vs trunk	63	0.94491	0.35268	0.3447	**no**	347	1200	1040
TPhP	trunk vs dashboard	34	-1.17169	0.24745	0.24132	**no**			
TiBP	dashboard vs seat	83	2.45677	0.0115	0.01402	yes			
TiBP	seats vs trunk	19	-2.30558	0.01854	0.02113	yes	139	22.1	100
TiBP	trunk vs dashboard	58	0.56695	0.57874	0.57075	**no**			
CDP	dashboard vs seat	15	-2.60795	0.00684	0.00911	yes			
CDP	seats vs trunk	51	0.0378	0.97051	0.96985	**no**	18	84.4	102
CDP	trunk vs dashboard	18	-2.38118	0.01469	0.01726	yes			
ip-TPP	dashboard vs seat	55	0.34017	7.39E-01	7.34E-01	**no**			
ip-TPP	seats vs trunk	57	0.49135	0.63053	0.62318	**no**	4.2	5.74	5.34
ip-TPP	trunk vs dashboard	56	0.41576	0.68421	0.67758	**no**			
TBP	dashboard vs seat	91	3.06151	1.05E-03	0.0022	yes			
TBP	seats vs trunk	25	-1.85203	0.06301	0.06402	**no**	79.2	14.1	26.7
TBP	trunk vs dashboard	65	1.0961	0.27986	0.27304	**no**			

Table S18 Analysis of published data on PBDEs in cars in ng/g. Studies included: [3–11]

PBDE	Number of studies	Median	Range (medians)		Countries represented						
			min	max	Czech	Germany	UK	USA	Kuwait	Pakistan	Nigeria
BDE 28	7	0.5	0.1	118	1		1	2	2	1	1
BDE 47	9	43	1.2	880	1	1	2	2	2	1	1
BDE 49	3	3	0.1	22.5	1			1			1
BDE 99	9	100	0.1	1130	1	1	2	2	2	1	1
BDE 100	8	16	0.1	211	1		2	2	2	1	1
BDE 153	8	13	0.3	320	1		2	2	2	1	1
BDE 183	9	9	0.8	73	1	1	2	2	2	1	1
BDE 206	5	1600	23.5	5200	1		1	1	1		1
BDE 207	4	920	17	4900	1		1	1	1		
BDE 208	4	940	7	4500			1	1	1		1
BDE 209	9	62000	168.5	280000	1	1	2	2	2	1	1

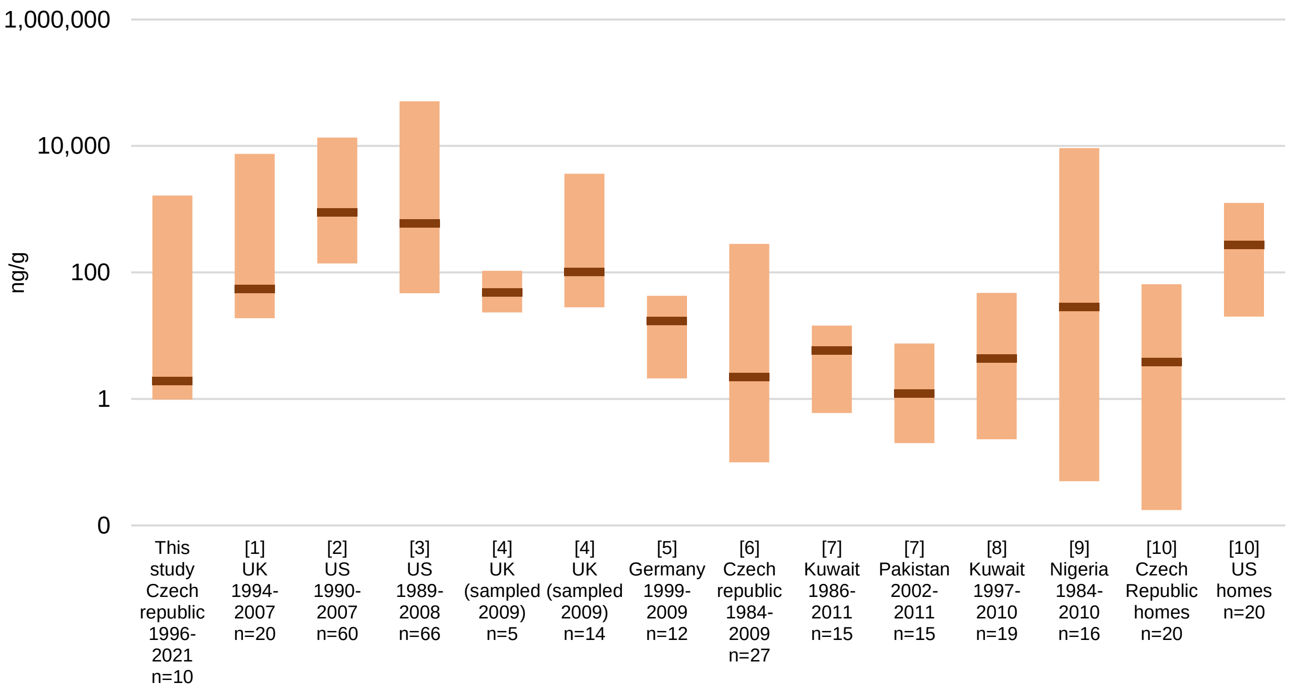

Figure S7 Literature values of BDE-47 in car dust, and a comparison with settled dust levels from US and Czech homes. Red lines indicate median, while the span of the orange bars indicates minima and maxima. The x-axis indicates the study, location, dates of vehicle manufacture, and number of vehicles/homes sampled (n). The following studies were included: 1: [11], 2: [10], 3: [9], 4: [7], 5: [4], 6: [8], 7: [3], 8: [5], 9: [6], 10: [12]

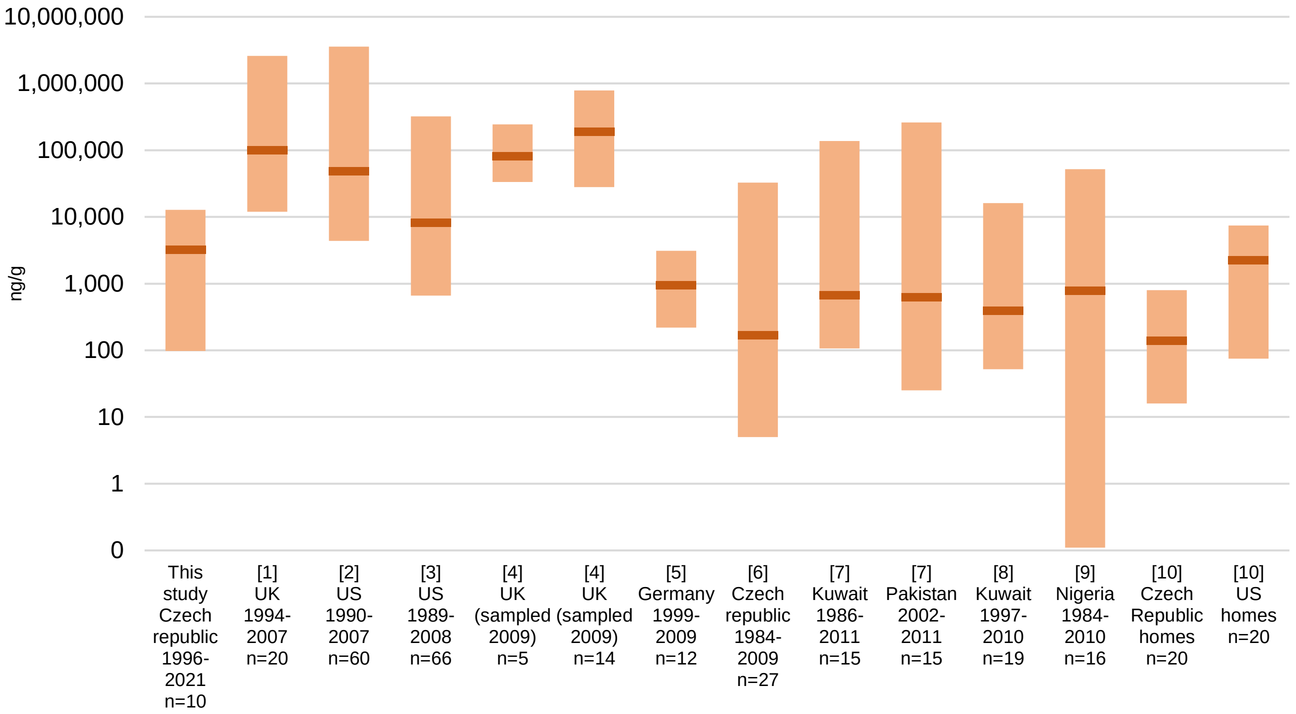

Figure S8 Literature values of BDE-209 in car dust, compared with US and Czech homes. Red lines indicate median, while the span of the orange bars indicates minima and maxima, The x-axis indicates the study, location, dates of vehicle manufacture, and number of vehicles/homes sampled (n). The following studies were included: 1: [11], 2: [10], 3: [9], 4: [7], 5: [4], 6: [8], 7: [3], 8: [5], 9: [6] , 10: [12]

Table S19 Ratio between BDE 209 and BDE 99

Car	Ratio
1996 Octavia	8
2001 Fabia	540
2002 Fabia	1079
2005 Fabia	126
2005 Octavia	30
2008 Octavia	3004
2009 Octavia	11640
2010 Fabia	54
2015 Fabia	197
2021 Octavia	958

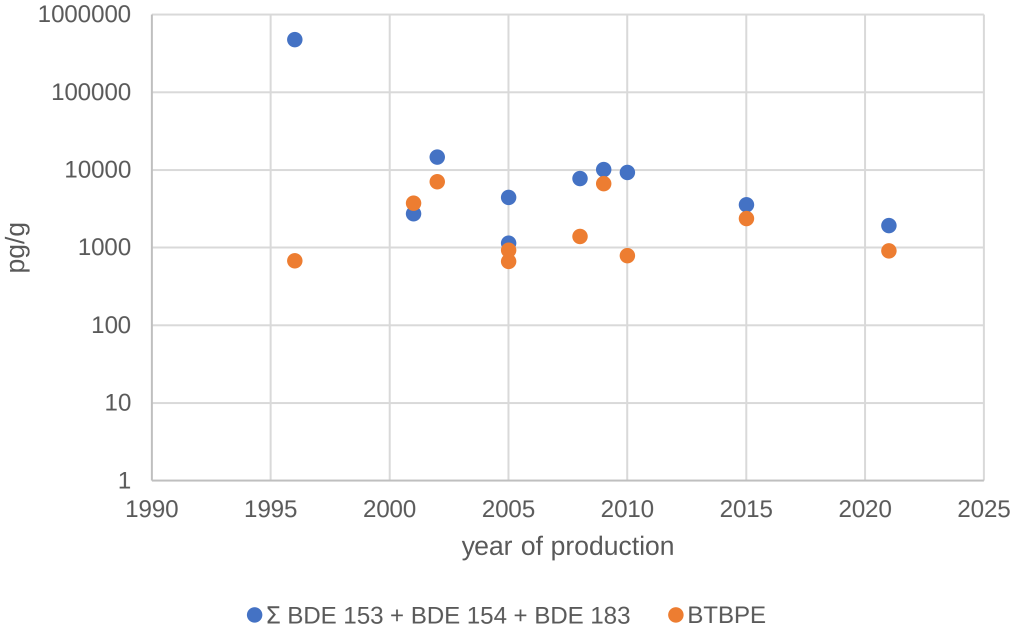

Figure S9 Concentrations of OctaBDE components compared with concentration of BTBPE. No indication of BTBPE being used as a replacement for octa-BDE in tested vehicles.

References

[1] Office of Reference Materials, Chemical Sciences Division, Certificate of Analysis SRM® 2585 Organic Contaminants in House Dust, National Institute of Standards and Technology: Gaithersburg, MD (2005). https://tsapps.nist.gov/srmext/certificates/2585.pdf (accessed July 2, 2024).

[2] NIST, Standard Reference Materials, NIST (2012). https://www.nist.gov/srm (accessed July 2, 2024).

[3] N. Ali, L. Ali, T. Mehdi, A.C. Dirtu, F. Al-Shammari, H. Neels, A. Covaci, Levels and profiles of organochlorines and flame retardants in car and house dust from Kuwait and Pakistan: Implication for human exposure via dust ingestion, Environment International 55 (2013) 62–70. https://doi.org/10.1016/j.envint.2013.02.001.

[4] S. Brommer, S. Harrad, N. Van Den Eede, A. Covaci, Concentrations of organophosphate esters and brominated flame retardants in German indoor dust samples, J. Environ. Monit. 14 (2012) 2482. https://doi.org/10.1039/c2em30303e.

[5] B. Gevao, F. Shammari, L.N. Ali, Polybrominated diphenyl ether levels in dust collected from cars in Kuwait: Implications for human exposure, Indoor and Built Environment 25 (2016) 106–113. https://doi.org/10.1177/1420326X14537284.

[6] S. Harrad, M.A.-E. Abdallah, T. Oluseyi, Polybrominated diphenyl ethers and polychlorinated biphenyls in dust from cars, homes, and offices in Lagos, Nigeria, Chemosphere 146 (2016) 346–353. https://doi.org/10.1016/j.chemosphere.2015.12.045.

[7] S. Harrad, M.A.-E. Abdallah, Brominated flame retardants in dust from UK cars – Within-vehicle spatial variability, evidence for degradation and exposure implications, Chemosphere 82 (2011) 1240–1245. https://doi.org/10.1016/j.chemosphere.2010.12.038.

[8] K. Kalachova, P. Hradkova, D. Lankova, J. Hajslova, J. Pulkrabova, Occurrence of brominated flame retardants in household and car dust from the Czech Republic, Science of The Total Environment 441 (2012) 182–193. https://doi.org/10.1016/j.scitotenv.2012.09.061.

[9] A.F. Lagalante, C.S. Shedden, P.W. Greenbacker, Levels of polybrominated diphenyl ethers (PBDEs) in dust from personal automobiles in conjunction with studies on the photochemical degradation of decabromodiphenyl ether (BDE-209), Environment International 37 (2011) 899–906. https://doi.org/10.1016/j.envint.2011.03.007.

[10] A.F. Lagalante, T.D. Oswald, F.C. Calvosa, Polybrominated diphenyl ether (PBDE) levels in dust from previously owned automobiles at United States dealerships, Environment International 35 (2009) 539–544. https://doi.org/10.1016/j.envint.2008.09.011.

[11] S. Harrad, C. Ibarra, M.A.-E. Abdallah, R. Boon, H. Neels, A. Covaci, Concentrations of brominated flame retardants in dust from United Kingdom cars, homes, and offices: Causes of variability and implications for human exposure, Environment International 34 (2008) 1170–1175. https://doi.org/10.1016/j.envint.2008.05.001.

[12] M. Venier, O. Audy, Š. Vojta, J. Bečanová, K. Romanak, L. Melymuk, M. Krátká, P. Kukučka, J. Okeme, A. Saini, M.L. Diamond, J. Klánová, Brominated flame retardants in the indoor environment — Comparative study of indoor contamination from three countries, Environment International 94 (2016) 150–160. https://doi.org/10.1016/j.envint.2016.04.029.
